# Beyond Exponential Decay: How Biphasic and Delayed Decay Dynamics Shape Marine eDNA Dispersal

**DOI:** 10.1002/ece3.72987

**Published:** 2026-01-25

**Authors:** Mohamed Yosri. Zanni, Verena M. Trenkel, Camille. Albouy, Ana C. Vaz, Claire B. Paris, Robin Faillettaz

**Affiliations:** ^1^ DECOD (Ecosystem Dynamics and Sustainability), Institut‐Agro, IFREMER, INRAe Nantes France; ^2^ Ecosystems and Landscape Evolution, Institute of Terrestrial Ecosystems, Department of Environmental Systems Science ETH Zürich Zürich Switzerland; ^3^ Swiss Federal Research Inst. WSL Birmensdorf Switzerland; ^4^ Instituto Nacional de Pesquisas Oceânicas (INPO) Rio de Janeiro Brazil; ^5^ Ocean Sciences, Rosenstiel School of Marine, Atmospheric, and Earth Science University of Miami Miami Florida USA; ^6^ DECOD (Ecosystem Dynamics and Sustainability), Institut‐Agro, IFREMER, INRAe Lorient France

**Keywords:** biphasic decay, decay, delayed decay, eDNA, Lagrangian particle tracking, monitoring, persistence

## Abstract

Accurate interpretation of marine environmental DNA (eDNA) monitoring surveys depends on understanding how eDNA concentrations change once released by organisms into the surrounding environment. Despite growing evidence for multiple eDNA decay patterns, such as biphasic and delayed decay, many studies assume by default a single‐phase exponential decay model. Here, we took a multiprong approach with a literature review of eDNA decay experiments, an experimental study of empirical decay patterns, and high‐resolution Lagrangian particle tracking to quantify how different decay patterns drive eDNA dispersal at two contrasted locations in the Bay of Biscay, Northeast Atlantic. Biphasic or delayed eDNA dynamic patterns were found in over half of the reviewed empirical studies and at 13°C and 20°C in our experiment, with the first 12 h being key to detecting biphasic or delayed decay dynamics. Compared to single‐phase exponential decay, the alternative decay dynamics increased eDNA concentrations by about a factor of two to four after 24 h, depending on the decay pattern. Assuming an exponential decay may lead to under‐ or overestimation of the amount of eDNA released when the decay dynamics are actually biphasic or delayed, depending on local hydrodynamics. In conclusion, considering the appropriate eDNA decay type is critical for marine eDNA transport models and eDNA monitoring data interpretation.

## Graphical abstract

We used a multiprong approach with a literature review of eDNA decay experiments, an experimental study of empirical decay patterns, and high‐resolution Lagrangian particle tracking to quantify how different decay patterns drive eDNA dispersal at two contrasted locations in the Bay of Biscay, Northeast Atlantic. Assuming an exponential decay may lead to under‐ or overestimation of the amount of eDNA released when the decay dynamics are actually biphasic or delayed, depending on local hydrodynamics.

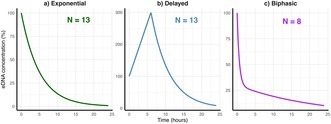

## 1 Introduction

The use of environmental DNA (eDNA) based approaches, including species‐specific approaches and metabarcoding, is increasingly popular for non‐invasive environmental monitoring (e.g., Takahara et al. 2012; Pawlowski et al. 2020; Fraija‐Fernández et al. 2020; Yao et al. 2022; Véron et al. 2023) and more generally for aquatic ecological studies (e.g., Rimet et al. 2025). The approach has been used for species detection and mapping (Nester et al. 2020), understanding species behavior (Takeuchi et al. 2019), deep‐water monitoring (Everett and Park 2018), and characterizing fish diversity and habitat preferences (Stoeckle et al. 2017). The term eDNA refers to total DNA obtained from environmental samples such as water, sediment, soil or air, and which includes DNA from various sources (unicellular, small multicellular organisms, tissue particles, gametes of multicellular organisms; Taberlet et al. 2012; Pawlowski et al. 2020). The two main fractions of eDNA are intracellular DNA (iDNA) contained in intact cells and extracellular DNA (exDNA; Nagler et al. 2022). Extracellular DNA is created from iDNA through cell lysis after cell death and active DNA extrusion by living organisms (de Ibáñez Alcoa et al. 2017). Following cell rupture, organelles such as nuclei and mitochondria may persist briefly before their own lysis, which releases additional exDNA into the environment (Heilig et al. 2023). The effects of factors such as microbial activity, presence of clay minerals, pH, UV, and temperature on cell lysis and exDNA degradation differ and depend on sample type (Nagler et al. 2022; Tzafesta and Shokri 2025; Brandão‐Dias et al. 2025). The state of exDNA fragments which can be free or bound to particles (Nagler et al. 2022) further influences their persistence (Barnes et al. 2014). In mesocosm trials with common carp, Turner et al. (2014) used sequential filtration to show that > 90% of newly released eDNA was retained on ≥ 1 μm filters, indicating it remained enclosed within cells or organelles (iDNA). The cell‐associated ‘protected’ fraction can persist for tens to over 100 h, whereas the dissolved exDNA fraction (0.2–0.45 μm) typically degrades within ~20–30 h (Zhao et al. 2021). The importance of particle size for degradation efficiency, with small eDNA particles (0.2–0.45 μm) degrading more rapidly than larger ones (1.0–10 μm), has been confirmed in various aquatic studies (e.g., Zhao et al. 2021; Brandão‐Dias et al. 2023; Brandão‐Dias et al. 2025). However, breakdown of large particles (e.g., cell‐associated or intracellular DNA) can generate smaller extracellular fragments, which may temporarily increase the apparent persistence of the small‐size fraction (Jo et al. 2021; Brandão‐Dias et al. 2023). As a result of different factors and processes, the decay of total eDNA may follow a multiphasic process (Harrison et al. 2019), which depends on the initial proportions of iDNA and exDNA, as well as their respective transformation rates in a given environment.

In marine environments, understanding the decay process of eDNA is particularly important as it is a key factor for the persistence and spatial transport of eDNA (Zanni et al. 2025). Understanding this process could help better describe biodiversity distributions and clarify the spatial footprint of eDNA data for quantitative approaches or small‐scale comparisons of presence‐absence biodiversity metrics (e.g., Rozanski et al. 2022; Macé et al. 2024). Unfortunately, restricted information on eDNA decay processes under natural conditions limits our ability to predict the true location and abundance of organisms from eDNA signals, increasing the risk of declaring both false‐positive range extensions and false‐negative non‐detections (Jo 2023; Snyder et al. 2023; Jo et al. 2025).

Over the past decade, decay rates of total eDNA have been quantified in controlled mesocosm or aquarium settings (e.g., Strickler et al. 2015; Sassoubre et al. 2016; Lance et al. 2017; Wood et al. 2020; Jo et al. 2020) as well as in a limited number of field studies (Collins et al. 2018; Sedlmayr and Schenekar 2024). As mentioned above, several environmental factors contribute to eDNA degradation in aquatic ecosystems, affecting both its stability and transport. In addition, water flow is multidirectional in marine systems and can vary strongly in space and time (e.g., tide, eddies, or storms). This makes the observation of eDNA decay dynamics in marine environments challenging and distinct from those in riverine systems and mesocosms.

Published empirical data suggests that, as expected due to the degradation processes summarized above, total eDNA degradation may not always be best described by a simple exponential decline. Instead, second‐order (biphasic or delayed) decay models might be more suitable to capture the complexity of the breakdown of eDNA fractions, particularly in marine environments where physical, chemical, and biological interactions could create distinct degradation phases (Eichmiller et al. 2016; Shogren et al. 2018; Allan et al. 2021; Jo 2023). A potential explanation of the delayed decay pattern is the delayed release of intracellular iDNA, particularly mitochondrial DNA, that might initially remain trapped within intact or partially degraded cells or adsorbed onto organic and inorganic particles, which provides physical protection against enzymatic degradation and renders the DNA temporarily undetectable by standard eDNA quantification techniques (Nagler et al. 2022; Brandão‐Dias et al. 2025). Over time, as cell membranes break down, these intracellular fragments could be released into the environment, resulting in a detectable rise in eDNA copy numbers during the early hours after shedding. An alternative–or complementary–explanation is that bacterial degradation could be inhibited (Brandão‐Dias et al. 2023). It has been suggested that iDNA and exDNA could undergo a pronounced initial‐rapid decay phase driven by bacterial enzymatic activity (Jo 2023). Accordingly, the presence or absence of a distinct early phase would likely reflect the intensity of bacterial activity, with exDNA being rapidly cleaved by microbial nucleases. For example, in mesocosm experiments with anchovy and sardine (Sassoubre et al. 2016), in temperature‐series trials with jack mackerel (Jo 2023), as well as in multi‐taxon tests at different temperatures ranging between 6°C and 23°C (Andruszkiewicz Allan et al. 2021), all observations concur with a ≥ 80% loss within the first 2 to 8 h. Environmental DNA may also sorb onto organic or inorganic particles, or become trapped in benthic biofilms; when these biofilms are later resuspended, they release the DNA back into the water column, creating a delayed secondary source (Snyder et al. 2023).

Despite accumulating evidence supporting the multiphasic nature of total eDNA degradation, many field studies have modeled the degradation process as a single‐phase exponential decay with constant decay rate (e.g., Thomsen et al. 2012; Collins et al. 2018). Similarly, early mesocosm studies in both marine (Thomsen et al. 2012) and freshwater (Barnes et al. 2014) systems adopted this approach. Following these foundational works, subsequent experimental studies applied the same single‐phase exponential decay model in different settings, including coastal seawaters (Collins et al. 2018), temperature‐controlled aquaria (Jo et al. 2019), and in multi‐species tank trials (Allan et al. 2021). Recent studies continued this trend. For example, a chytrid/ranavirus decay study reported half‐lives of 19–52 h using a simple exponential model (Trafford et al. 2024), while flow‐flume tests on eDNA transport likewise modeled decay with a first‐order constant decay rate (Dercksen et al. 2025). Scriver et al. (2023) found in their review of 20 recent studies that all decay rates were ultimately expressed with a first‐order model, reflecting the widespread use of the single‐phase model without considering alternative formulations. However, in certain cases, it could oversimplify the underlying processes, which are increasingly recognized as multiphasic and thus more complex (McCartin et al. 2022; Jo 2023). Ignoring this could have impacts on the interpretation of eDNA field observations, though this has not been studied so far. Particle‐tracking simulation approaches have become widely used in marine eDNA research because they permit evaluating how uncertainties in decay dynamics, shedding rates, and hydrodynamic transport propagate to eDNA dispersal (e.g., Andruszkiewicz et al. 2019; Fukaya et al. 2020; Pastor Rollan et al. 2024; Zanni et al. 2025).

In this study, we carried out a literature review to summarize the empirical evidence for single‐phase and two‐phase eDNA decay, and an experimental study investigating the potential interplay between water temperature and decay dynamics. We then applied a Lagrangian modeling approach to examine how a biphasic and delayed decay could affect the dispersal of eDNA particles and hence their potential detectability in marine field studies compared to the traditional single‐phase exponential decay.

## 2 Materials & Methods

### 2.1 Review of Observed eDNA Decay Processes

To appraise the importance of biphasic and delayed decay processes in marine studies, we conducted a review of eDNA decay processes in the published literature. We considered all potential studies, even when the original authors did not explicitly model or discuss a biphasic or delayed decay. We assessed peer‐reviewed studies that experimentally measured eDNA decay rates and dynamics using time‐series data collected under controlled or in situ conditions. We started from the decay‐rate dataset compiled by Scriver et al. (2023), then expanded it by screening Google Scholar and Web of Science for relevant publications that studied eDNA decay in aquatic environments. From this initial list of studies, we then selected only studies that presented decay time‐series data with sampling points during the early phase (< 12 h). This threshold was chosen to ensure that the selected studies contained measurements within the first hours after collection, where non‐exponential behaviors typically occur. Studies that sampled only at *t* = 0 and then again ≥ 12 h later were excluded because they did not capture early‐phase dynamics. This provided 40 relevant studies which were visually screened to categorize them into exponential, biphasic, or delayed decay, see dataset provided in Table S1.

### 2.2 Experimental Decay Process Study

#### 2.2.1 Experimental Setup

To refine the knowledge on the early decay phase, we conducted an experiment in April 2021 keeping water with eDNA shed by European seabass ( *Dicentrarchus labrax* Linnaeus, 1758) at three temperatures: 13°C (temporal variation 12.7°C–14.9°C), 20°C (18.9°C–23.5°C), and 27°C (25.8°C–29.5°C). For this, 120 L of water were collected from a scientific aquaculture facility at Ifremer Brest, which contained only seabass and was at ambient temperature at 13°C. The water in the aquaculture tank comes directly from the Bay of Biscay after sand filtering and is renewed every hour. The sampled water was then distributed into 20‐L tanks, resulting in two replicates (A and B) per temperature (2 × 20 L). The temperature was achieved and maintained using immersion heaters that were decontaminated with bleach before the experiment and rinsed twice with ultra pure water. Between the start of the experiment (*t* = 0 h) and the end (*t* = 26.5 h), seven 2‐L samples were filtered from each tank using 0.8 μm Sylphium filter capsules around time *t* = 0, 0.5, 1, 2, 4, 6, 23, 26 h. To perform the filtration, we used an Athena peristaltic pump (Proactive Environmental Products LLC; nominal flow of 1.1 L/min), which prevented any contact between the water and the filtration device. Separate disposable sterile tubes were used for each filtration capsule. At the end of filtration, the water inside each capsule was emptied and replaced with 5 mL of CL1 conservation buffer. Then we stored the capsules at room temperature (Polanco Fernández et al. 2020). To avoid contamination, all sampling steps were carried out using disposable gloves and single‐use filtration equipment. The filters were analyzed by a specialized forensic DNA laboratory (SINSOMA). A total of 75 samples were extracted and analyzed using droplet digital PCR (ddPCR) with a primer specifically developed for seabass (Trenkel et al. 2025). Three technical replicates of 6.5 μL were analyzed per filter. For the full molecular analysis protocol, see Trenkel et al. (2025).

#### 2.2.2 Decay Process Model Fitting

To determine the most suitable model representing eDNA decay at different temperatures (13°C, 20°C, 27°C), two decay models were fitted to the experimental eDNA data from the European seabass experiment. First, an exponential first‐order decay model (Barnes et al. 2014):

(1) $$C\left(t\right)=C{\left(0\right)}_{0}\times e^{-k\times t}$$

where $C{\left(0\right)}_{0}$ is the initial concentration (copies·μL^−1^), *k* is the constant decay rate per hour and *t* is the time from eDNA shedding (in hours). Second, a second‐order decay model (Brouwer et al. 2017):

(2) $$C\left(t\right)=C{\left(0\right)}_{1}\times e^{-k_{1}\times t}+C{\left(0\right)}_{2}\times e^{-k_{2}\times t}$$

where $C{\left(0\right)}_{1}$ and $C{\left(0\right)}_{2}$ are the initial concentration (copies·μL^−1^) of the two model components and $k_{1}$ and $k_{2}$ the corresponding decay‐rate constants (per hour). The advantage of this mixture model is that it can represent both biphasic and delayed decay, does not require specification of the duration of each phase, the transition from the first to the second phase is smooth, and it has only four parameters.

The two models were fitted as nonlinear mixed‐effects models to the replicate‐level concentration data, using the *nlme* R package (Pinheiro et al. 2023). Each technical replicate was treated as an individual observation. Temperature (13°C, 20°C, 27°C) was included as a fixed effect for all model parameters, C_0_ and k for the exponential model, and C_01_, k_1_, C_02_, k_2_ for the second‐order model. Biological replicate identity (“A” and “B”), combined with the temperature treatment, defined the random‐effects grouping variable. The two model types were compared using Akaike's Information Criterion (AIC) and the coefficient of determination (*R* ^2^). The best fitting model was interpreted as providing evidence for the underlying decay process (first or second order dynamics).

### 2.3 Lagrangian Modeling

#### 2.3.1 Lagrangian eDNA Model Setup

**FIGURE 1:**
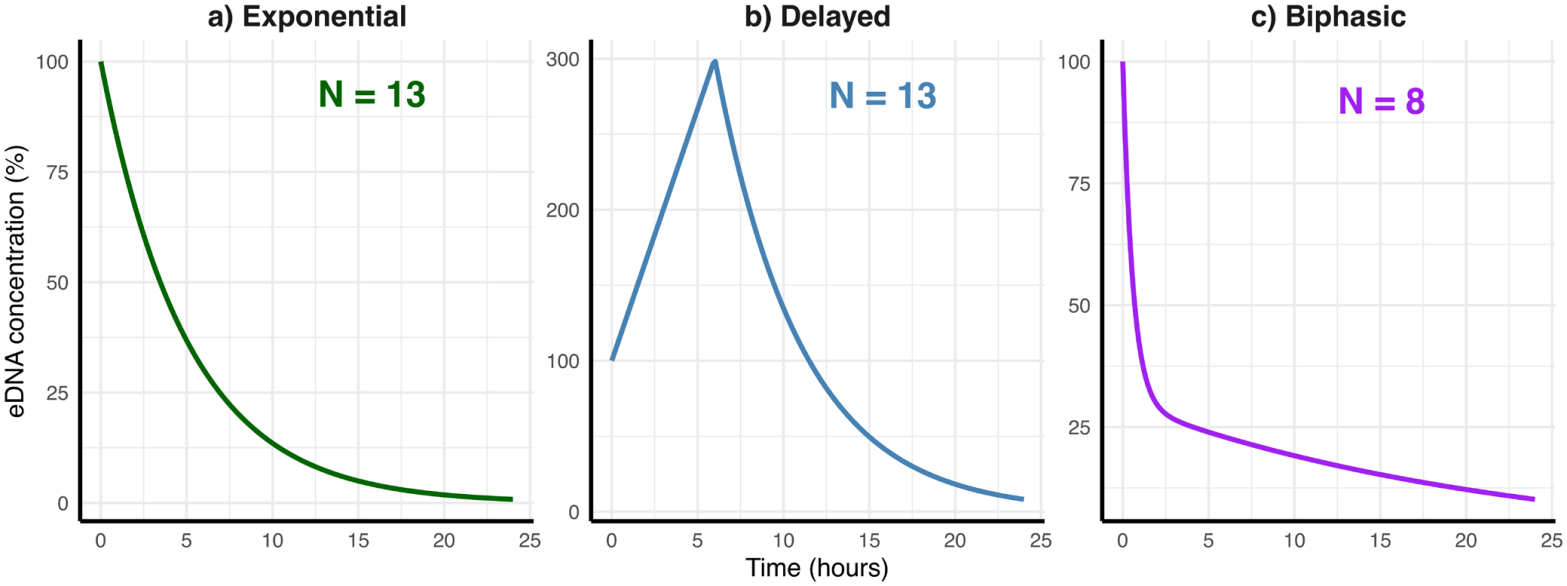
Schematic representation of functional forms describing eDNA concentration dynamics identified in the literature review, with the number of studies (N) that displayed each decay process.

To explore the impact of different decay functional forms on eDNA particle densities, we implemented four decay process scenarios in the Lagrangian eDNA transport model developed by Zanni et al. (2025) for the Bay of Biscay, using the Connectivity Modeling System (CMS; Paris et al. 2013). Three of the decay process scenarios were based on the literature review (sections 2.1 and 3.1) and on the experimental study (sections 2.2 and 3.2). They included exponential, biphasic, and delayed decay (Figure 1, Table 1). A no‐decay scenario was implemented for comparison. The same temperature‐dependent exponential decay relationship was used for all decay scenarios as the unique function in the exponential case and approximately in the second phase for biphasic and delayed decay functions. This exponential function corresponds to the “moderate” decay rate defined in Zanni et al. (2025), which represents the median decay–temperature relationship derived from a bibliographic review of teleost fish eDNA decay rates and is defined as:

(3) $$k=0.0419\times e^{\left(0.0776\times \text{Temperature}\right)}$$

here, *k* represents the probability of decay: at each timestep, every individual eDNA particle either decays or remains present, following a binomial survival process. In CMS, the horizontal diffusivity coefficient was set to *K* = 1 m^2^ s^−1^ as derived from Okubo (1971) to match the 500‐m grid resolution of the hydrodynamic dataset (section 2.3.2). Particle trajectories were integrated using a classical fourth‐order Runge–Kutta scheme with a fixed 3‐min time step, balancing numerical stability and computational efficiency on the high‐resolution mesh used.

**TABLE 1:** Summary of the four decay scenarios simulated in the CMS. The decay scenario and half‐lives were defined based on the literature review (Results section 3.1) and the experimental study (Results section 3.2).

Decay scenario	Description	Half‐life (h)
Delayed	Delayed decay with an initial increasing phase: eDNA counts triple within the first 6 h, then decline exponentially	19.9
Biphasic	Biphasic decay with a rapid early decrease, followed by a much slower second phase	0.9
Exponential	Exponential particle decay	6.4
No‐decay	Released particles remain intact; no decay is applied	∞

To implement the biphasic and delayed decay scenarios within the Lagrangian framework, where eDNA is represented as discrete particles, we used two complementary approaches. For the delayed decay scenario, we implemented a custom pre‐ and post‐processing routine to reproduce random particle division. During the first 6 h after release, 20% of the particles were randomly selected each hour and divided (duplicated) at their exact positions, resulting in a threefold increase in particle numbers 6 h after release. The threefold increase was chosen as it corresponds to the order of magnitude of the pattern reported in several empirical studies and in our own experimental observations. After 6 h, no further division occurred, and all particles (original and divided) only decayed and were tracked until the end of the simulation. CMS only handles one constant decay rate per particle group. For the biphasic decay scenario, we therefore split each set of released particles into two groups, each experiencing a distinct constant decay rate throughout the simulation. We tested several combinations of decay rates and proportions of fast‐ versus slow‐decaying particles to identify the setup that most closely reproduced the typical biphasic pattern described in the literature. The selected setup consisted of assigning a fast decay rate of 0.50 h^−1^ to 70% of the particles and a slower decay rate of 0.013 h^−1^ to the remaining 30%.

#### 2.3.2 Hydrodynamic Data

**FIGURE 2:**
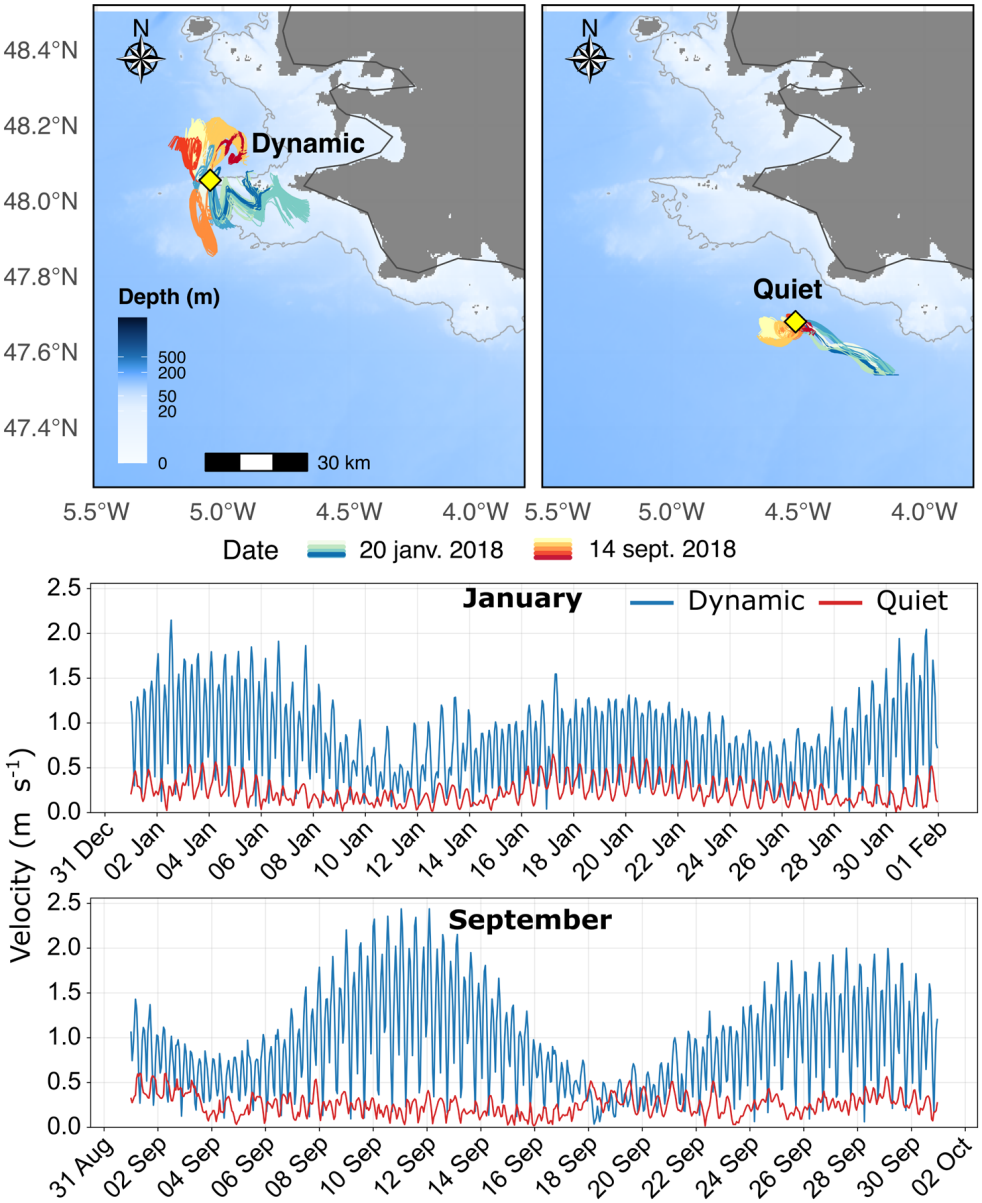
Map of the study region (Brittany, France) showing the two particle‐release locations used in the Lagrangian simulations: Dynamic (site 1) (48.05° N, 5.05° W; bottom depth 50 m), and quiet (site 2) (47.68° N, 4.51° W; bottom depth 66 m). Example particle trajectories are shown for both sites over 2 days, colored by hour of the day. All particles were released at the sea surface (Top panel). Hourly surface current velocity at the two release sites in January and September 2018 (Bottom panel) from the high‐resolution implementation of MARS3D hydrodynamic model in the Bay of Biscay Zone described in Caillaud et al. (2016).

The CMS was used to release and follow particles at the surface from two coastal locations at the tip of Brittany in the Bay of Biscay, northeast Atlantic (Site 1: 48.05° N, 5.05° W, bottom depth 50 m, and Site 2: 47.68° N, 4.51° W, bottom depth 66 m). Site 1 is characterized by strong tidal currents (0.01–2.4 m s^−1^) and is hereafter referred to as the *dynamic site*, while Site 2 is exposed only to weaker water flows (0.01–0.6 m s^−1^; Figure 2), hereafter the *quiet site*. These contrasted sites enabled evaluation of the impact of different decay functional forms on particle transport and density.

Hydrodynamic forcing for the two locations was derived from the MARS3D model coupled with the AGRIF nesting library (Caillaud et al. 2016), configured at a 500‐m spatial resolution and hourly temporal frequency over the English Channel–Bay of Biscay domain (Caillaud et al. 2016). Model fields were re‐gridded to meet the CMS input specifications; sigma‐coordinate layers were interpolated onto fixed‐depth (Z) levels, and the horizontal data were remapped from an Arakawa C‐grid to an Arakawa A‐grid. The hydrodynamic variables used in our 2D simulations included potential seawater temperature, as well as eastward (u) and northward (v) components of ocean current velocities.

#### 2.3.3 Particle Release

To account for both seasonal and short‐term temporal hydrodynamic variability (currents and temperature), we performed the CMS particle tracking releases every 2 h throughout the entire months of January and September 2018, representing a total of 732 releases per scenario. To ensure numerical convergence, for each event and scenario, we released 10,000 particles at the surface of the two sites (i.e., dynamic and quiet; Figure 2) and tracked them over a 24‐h period. The chosen particle release number corresponds roughly to biologically realistic shedding rates from a single fish as found in experimental studies (Andruszkiewicz Allan et al. 2021). The 24‐h time window was selected as, within this period, most particles have decayed (half‐lifes in Table 1). This simulation design resulted in a total of 29.28 million particles tracked from 2928 release events.

The two‐month release schedule (January and September 2018) spanned two complete spring–neap cycles (circa 14.8 days per cycle, 4 cycles in total; Kvale 2006), covering tidal coefficients from neap values below 30 up to equinoxial spring values above 110 (please note that tidal coefficients illustrate the tidal intensity, but are only used in France). Because the tidal coefficient is simply a normalized proxy for the daily tidal range, driven by the spring–neap oscillation of lunar–solar alignment (El Tawil et al. 2019), our schedule ensured that the simulations embraced almost the full tidal intensity spectrum encountered on the French Atlantic coast in a typical year. Releasing particles every 2 h also guaranteed that each semi‐diurnal phase, from high water to peak flood, was explored, giving a realistic picture of eDNA advection and dilution under both astronomical (spring–neap) and intra‐tidal (12 h 25) variability (Velímský et al. 2018).

#### 2.3.4 Dispersal Evaluation Metrics

To summarize and explore our simulation results, we applied an event‐based metrics framework as in Zanni et al. (2025), with additional refinements to account for the impact of the decay scenario. We computed several dispersal metrics to characterize the spatial and temporal behavior of the simulated particles. These metrics were calculated using the geographic positions of all released particles (*i* = 10,000) recorded at an hourly time interval (*t* = 0 to *t* = 24 h) for each release event (*j* = 2928) and scenario (*s* = 4). Then we calculated six metrics: the number of particles alive, their center of mass, their mean distance from release locations, their dispersion relative to the center of mass, the convex‐hull area covered by them and the density of eDNA particles within the convex‐hull. The number of particles alive ${\mathrm{Np}}_{\mathrm{sjt}}$ represents the total number of particles remaining in the simulation at a given time step t for release event *j* and scenario *s*. A particle is considered “alive” if it has not exited the domain or decayed. The center of mass is the mean geographic position (${\underline{x}}_{\mathrm{sjt}}$, ${\underline{y}}_{\mathrm{sjt}}$) of particles of release *j* at time *t*. It is calculated separately for longitude and latitude using the arithmetic mean of all particle coordinates, such as:

(4) $${\bar{x}}_{\mathrm{sjt}}=\frac{1}{N_{t}}\sum_{j=1}^{2928}\sum_{i=1}^{{\mathrm{Np}}_{\mathrm{jt}}}x_{\text{sjit}},{\bar{y}}_{\mathrm{sjt}}=\frac{1}{N_{t}}\sum_{j=1}^{2928}\sum_{i=1}^{{\mathrm{Np}}_{\mathrm{jt}}}y_{\text{sjit}}$$

where $x_{\text{sjit}}$ and $y_{\text{sjit}}$ are the coordinates of particle *i* from event *j* at time *t* for scenario *s* and ${\mathrm{Np}}_{\mathrm{sjt}}$ is defined above.

The mean distance ($L_{\mathrm{sjt}},$ in km) quantifies how far, on average, the particles became displaced from the initial release location. It is calculated as the great‐circle (haversine) distance between the release location (*x*_*0*_, *y*_*0*_) and the center of mass of particles from release *j* at a time *t* using the *geosphere* R package (Hijmans 2025). The dispersion ($D_{\mathrm{sjt}}$, in km^2^) measures of how widely the particles of each release event became dispersed around their center of mass at time *t*. It is quantified by the variance of the distances of individual positions from the center of mass, as:

(5) $$D_{\mathrm{sjt}}=\frac{1}{N_{\mathrm{sjt}}}\sum_{i=1}^{N_{t}}{r_{\text{sjti}}}^{2}$$

where $r_{\text{sjti}}$ is the haversine distance (in km) of particle *i* from the center of mass of all particles of event *j* at time *t* for scenario *s*. A large value indicates that the particles became widely dispersed, while a low value indicates that they remained concentrated around the center of mass.

The convex‐hull area ($A_{\mathrm{sjt}},$ in km^2^) represents the surface of the smallest convex envelope that encloses all particles of event *j* at time *t*. It is computed by determining all outer‐edge particles (the hull vertices) and summing the triangle areas formed by successive vertex pairs, thereby accounting for every corner of the polygon. A large value indicates that the particles have spread out widely, while a small value means the particles are still tightly clustered.

Finally, the density ($\rho_{\mathrm{sjt}}$, in particles per km^2^) quantifies how tightly clustered particles are within the convex‐hull area of each event *j* at time step *t* for scenario *s*. It is computed by dividing the number of particles (${\mathrm{Np}}_{\mathrm{sjt}}$) still alive at time *t* by the area of the convex‐hull enclosing their positions, such as:

(6) $$\rho_{\mathrm{sjt}}=\frac{{\mathrm{Np}}_{\mathrm{sjt}}}{A_{\mathrm{sjt}}}$$

A higher value of $\rho_{\mathrm{sjt}}$ indicates a compact and aggregated particle cloud, whereas a lower value suggests widespread dispersion across a larger area.

#### 2.3.5 Statistical Analyzes

To test for differences among the four decay scenarios (biphasic, delayed, exponential, no‐decay) using the output dispersal evaluation metrics (mean distance, dispersion, convex‐hull area, density) over the first 24 h after release, for each release site, we first standardized each metric by centering and scaling metrics for each scenario and release site to ensure comparability. We then fitted a three‐way mixed‐effects ANOVA using the *lme4* R package (Bates et al. 2015), with fixed effects for scenario (4 levels), dispersal metric (4 levels), time since release in hours (24 levels), all two‐ and three‐way interactions, and a random intercept for release event. Time since release was treated as a factor to account for non‐linear temporal dynamics. Type III F‐tests were carried out to test the significance of all explanatory factors and interactions using the ANOVA method (lmerTest, Kuznetsova et al. 2017). We estimated partial η^2^ effect sizes for each explanatory factor (scenario, metric and time) with the effect size R package (Ben‐Shachar et al. 2020). Significant interactions were explored by testing pairwise contrasts of estimated marginal means via the *emmeans* R package (Lenth 2023). All statistical analyzes were conducted in R (R Core Team, 2025).

## 3 Results

### 3.1 Review of Observed eDNA Decay Processes

Among the 40 studies reviewed, 36 collected multiple early time‐points immediately after water collection, typically within the first 0–12 h. In less than half of these studies (36%, *n* = 13) a simple exponential decay of eDNA was identified (Figure 1a), while in the remaining studies two distinct decay patterns were visible, with around two thirds of them (62%, *n* = 13) showing delayed decay characterized by an initial rise in the number of eDNA copies, followed by an exponential decreasing phase (Figure 1b). In these studies, eDNA copy numbers increased several fold during the first 5–6 h of the experiment. The second observed pattern was biphasic decay (38%, *n* = 8), consisting of an initial rapid decline in the number of eDNA copies followed by a slower decreasing phase (Figure 1c).

### 3.2 Experimental Decay Process Study

**FIGURE 3:**
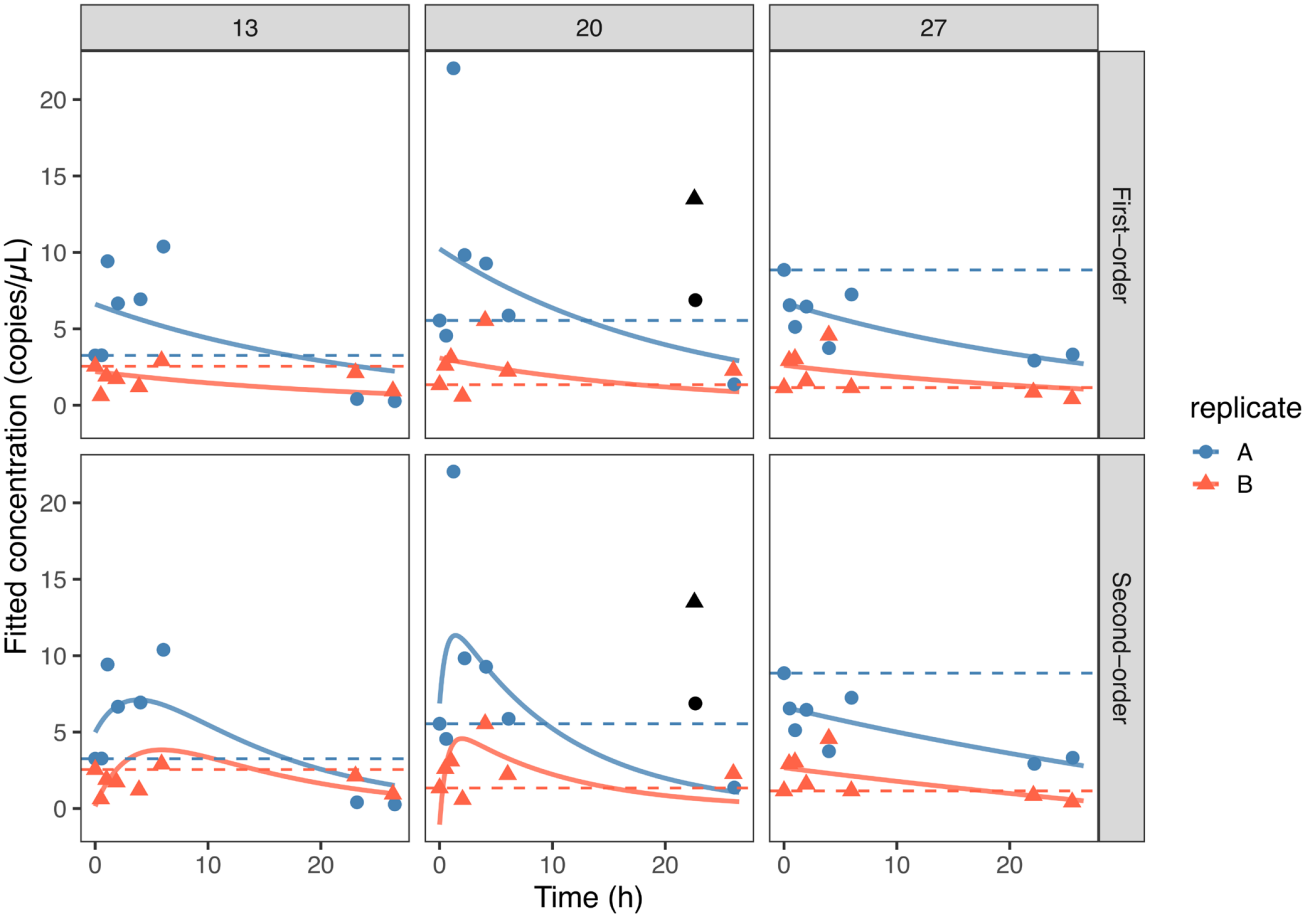
Data (dots and triangles) and fitted first and second‐order models (solid lines) for each experimental temperature and experimental replicate. The black dots mark outliers that were excluded from model fitting.

In our experimental dataset, we consistently observed a delay phase in eDNA decay across all temperatures (13°C, 20°C, and 27°C) and in both biological replicates (A and B), except for replicate A at 27°C (Figure 3). The measured eDNA concentrations rose during the first 0–6 h (Figure 3). In all experimental conditions, a subsequent clear decline towards very low eDNA copy numbers by the end of the experiment was found. Unexpectedly, replicate A consistently showed higher eDNA concentrations than replicate B for all three temperatures (Figure 3), even though decay trends were similar.

The first and second order models provided broadly similar fits, as indicated by their AIC values and $R^{2}$ scores (Table S2). Upon closer inspection, the concentrations measured after 22 h in the 20°C tanks appeared unusually high. In the initial fit including this time point, the first‐order exponential model produced identical decay‐rate constants of 0.04 h^−1^ at 13°C and 27°C, but yielded a zero (*k* = 0) estimate at 20°C (Figure S1, Table S2). We therefore refitted both models after entirely removing the 22 h time point (Figure 3). After refitting, the exponential model (Equation 1) estimated decay rate constants of 0.04 h^−1^ at 13°C, 0.05 h^−1^ at 20°C, and 0.03 h^−1^ at 27°C, suggesting only weak temperature dependence. The AIC value for this reduced dataset was smaller for the second‐order formulation (695 compared to 709 for first‐order model), indicating that this model better described the data. The $R^{2}$ values ranged between 0.59 and 0.48 for the second and first order model respectively, highlighting the unexplained variability. Overall, the second‐order model reproduced the observed delayed decay, with an early increase in concentration followed by a sharper decline (Figure 3). For 13°C and 20°C, the fitted decay phase exhibited decay rate constants of 0.08 and 0.10 h^−1^, respectively, substantially higher than the first‐order rates. In contrast, at 27°C the second‐order model did not distinguish two phases: the fitted curve was essentially identical to the single‐phase exponential model (Table S2).

### 3.3 Lagrangian eDNA Modeling

#### 3.3.1 Main Drivers of Particle Dispersal

**FIGURE 4:**
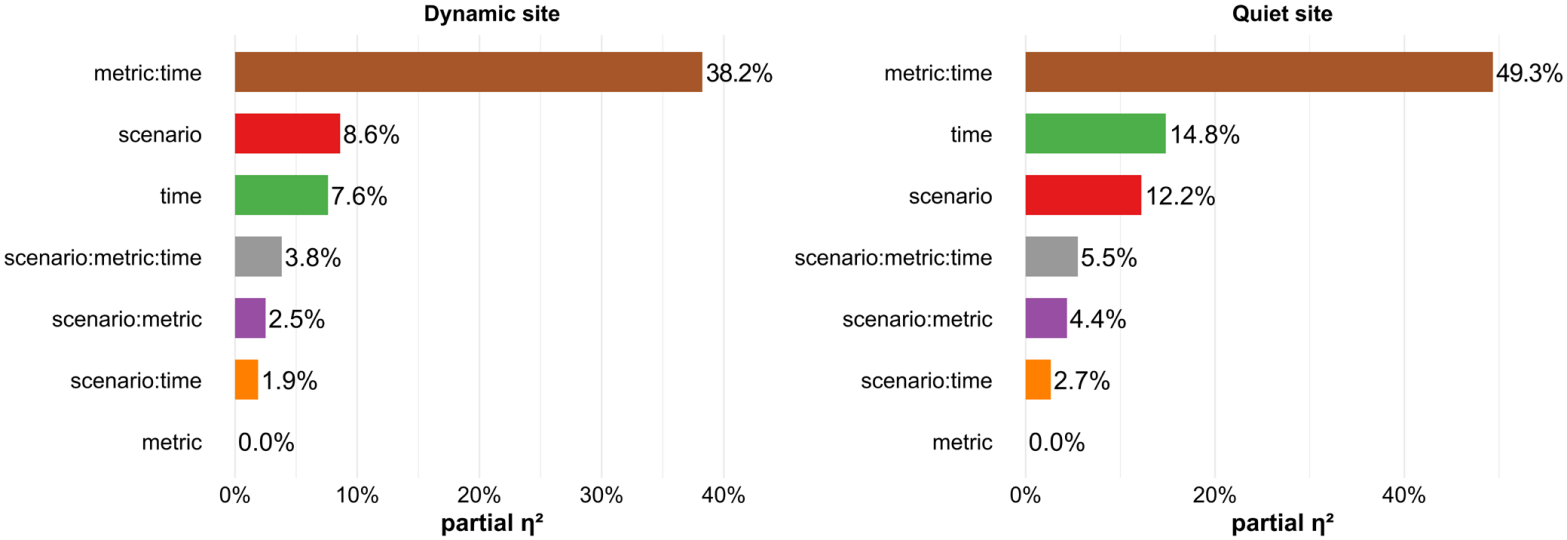
Relative contributions (i.e., effect size η^2^ from three‐way mixed‐effects ANOVA) of decay scenario, metric, time since release and their interactions to explain the variance in standardized evaluation metrics over the 24‐h dispersal from the dynamic site (left panel) and the quiet site (right panel).

To quantify how decay scenario, time since release, and dispersal metric jointly influenced the dispersal outcomes (including interactions), we fitted a three‐way mixed‐effects ANOVA using the standardized metric values (mean distance, dispersion, density, and convex‐hull area) as the response. All four metrics were analyzed together within the same model, with “metric”, decay scenario, time since release and their interactions as fixed effects, and release event as a mixed effect. The most important factor was the interaction term between all dispersal metrics and time since release for both locations: 38.2% of the variance for the dynamic site, which was subject to strong tidal currents, and 49.3% for the quiet site, characterized by weak hydrodynamism (Figure 4). This indicates that each metric followed its own characteristic diurnal course. The effect of scenario ranked second for the dynamic site and third for the quiet site, explaining 8.6% and 12.2% of variance, respectively, while the main effect of time since release accounted for 7.6% for the dynamic site and 14.8% for the quiet site. Higher‐order interactions were comparatively weak (≤ 5.5%; Figure 4). Considering all terms involving the decay scenario, the type of decay function used explained 16.8% of variance for the dynamic site and 24.8% for the quiet site. Thus, variation in the daily profiles of the four dispersal metrics dominated the overall structure of the results. Lastly, unaccounted environmental variability due to differences between January and September and variations along the tidal cycle were responsible for 37.4% of residual variance for the dynamic site, approximately twice the effect of decay function type, but only 11.1% for the quiet site.

#### 3.3.2 Horizontal Spread of Particles

**FIGURE 5:**
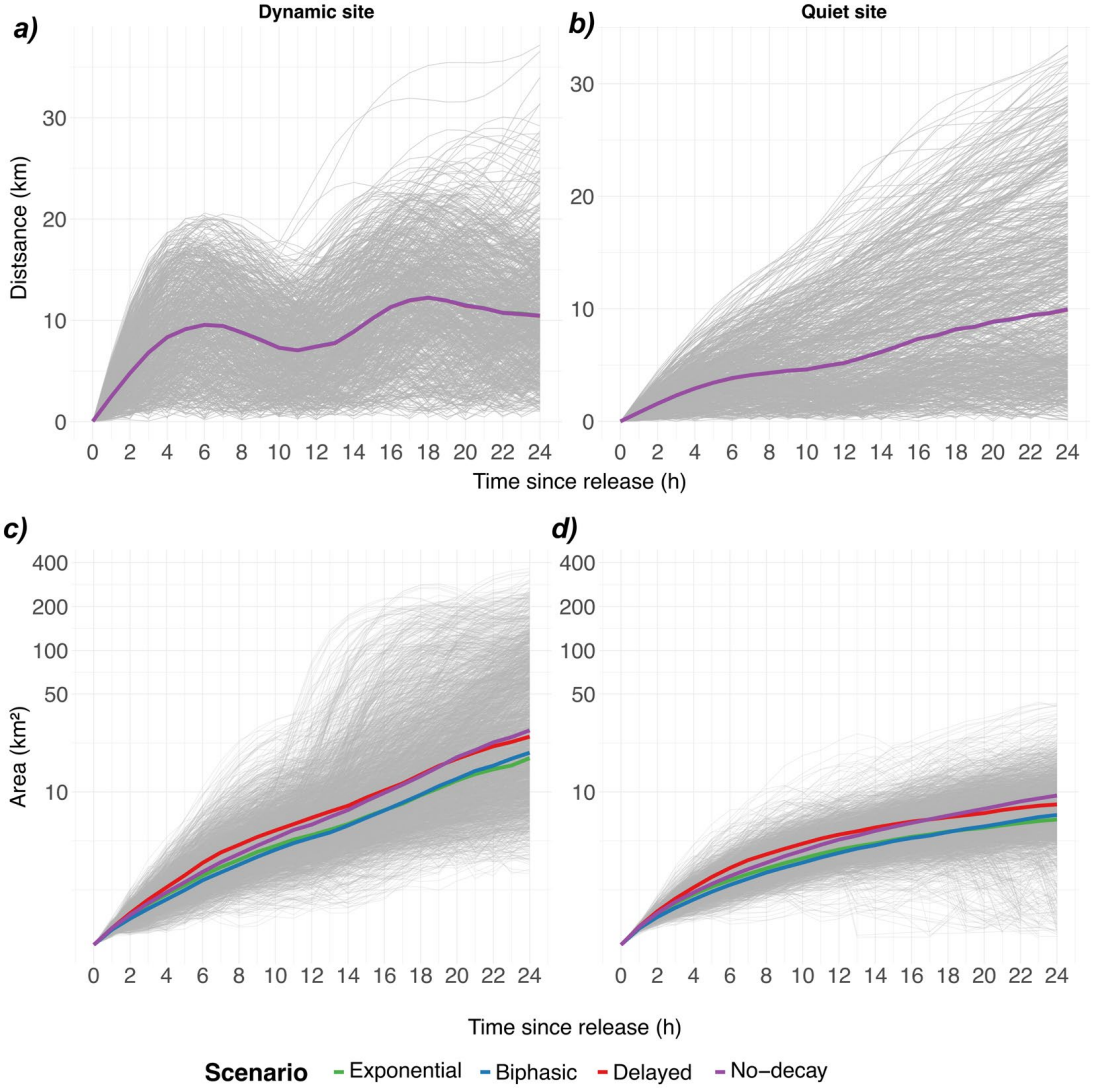
Horizontal dispersal of the particles in 24 h (a, b) Distance traveled from release (c, d) Convex‐hull area (log‐transformed Y axis), with graylines corresponding to median values of individual release events all scenarios combined, colored curves show the scenario‐specific medians (Dynamic site, left panels; Quiet site, right panels).

Within each release site, as expected, the particles followed the same mean trajectory for all four decay‐process scenarios (Figure 5a,b). For the dynamic site, the median of mean distances from the release location reached about 9 km after 6 h, then retreated by approximately 2 km for times since release between 7 and 10 h, before rising again to a maximum of circa 12.5 km after 18 h. By the end of the 24‐h period, the median of the centers of mass was situated at circa 10 km from the release location. At the quiet site, the median of mean distances grew steadily from 0 km at release to roughly 10 km after 24 h, showing only weak tidal influence. Inter‐event variability was substantial at both sites, though higher at the quiet site (Figure 5b).

The convex‐hull area (km^2^) encompassing all particles still alive increased monotonically with time in all decay scenarios and for both release sites, illustrating that particles continually dispersed from their release locations (Figure 5c,d). The area increased rapidly during the first 6 h to reach 2 km^2^ at the dynamic site and 0.4 km^2^ at the quiet site, then continued to grow gradually through 24 h up to around 27 km^2^ at the dynamic site, while reaching only 9 km^2^ at the quiet site. Across all simulations, the convex‐hull area at the dynamic site was consistently larger than at the quiet site, and maximum inter‐event variability at the dynamic site was an order of magnitude higher (Figure 5c, gray lines). Although the median of trajectories by event (gray lines, Figure 5c,d) varied greatly in both pattern and magnitude, the scenarios' median trajectories followed similar trends. Only the delayed decay and no‐decay scenarios spread over a marginally wider area due to the larger number of particles for both scenarios, while the exponential and biphasic decay scenarios spread over very similar areas (Figure 5c,d).

#### 3.3.3 Effect of Decay Process on Particle Density

**FIGURE 6:**
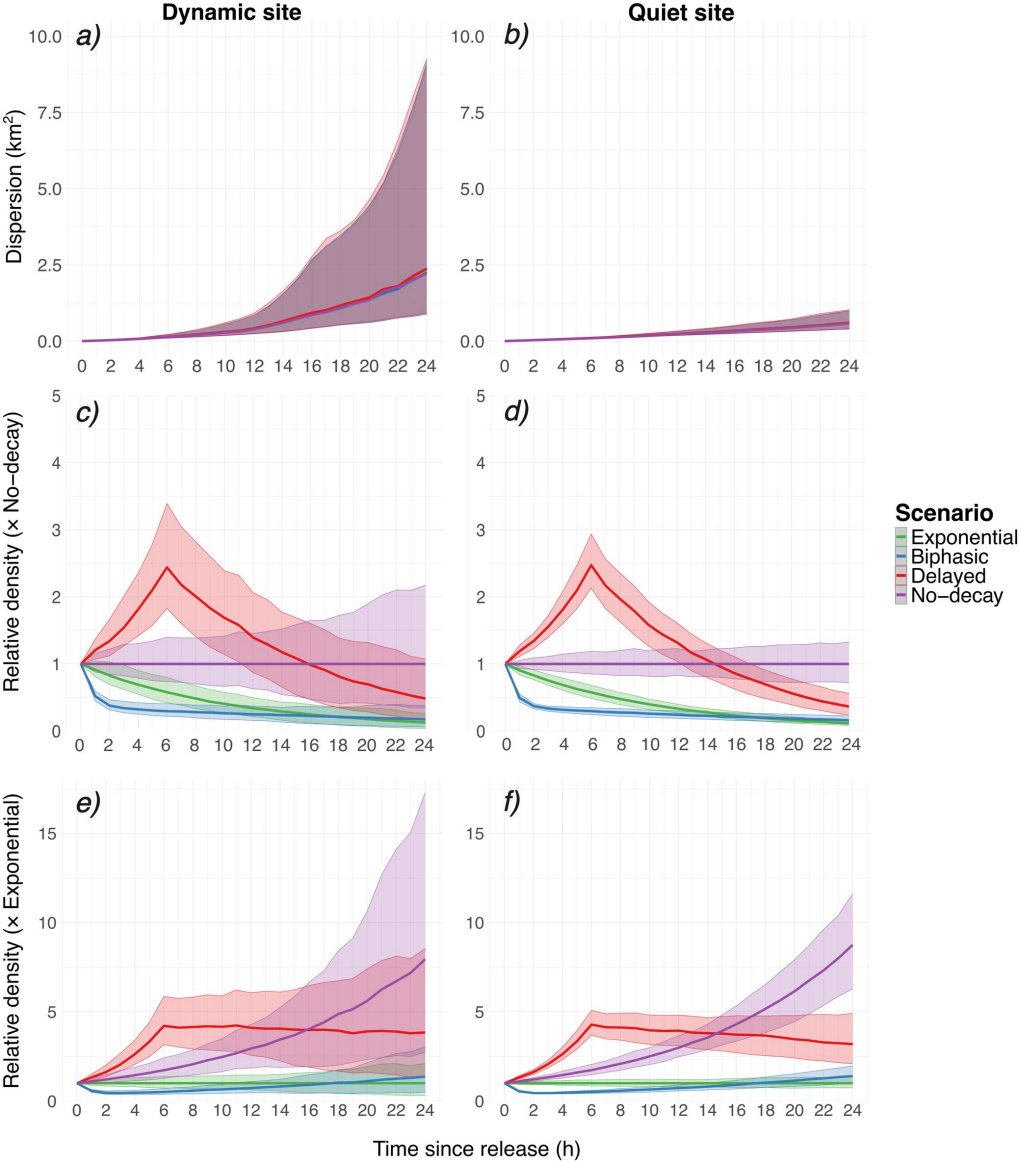
(a, b) Dispersion. (c, d) Relative density normalized to the no‐decay median. (e–f) Relative density normalized to the exponential‐decay median. In all panels, ribbons show the 20%–80% percentile band and lines show the median for each decay scenario. Left panels: Dynamic site; right panels: Quiet site.

During the first 5 h after release, particle trajectories remained tightly clustered, leading to little dispersal among decay scenarios at the dynamic site (Figure 6a). At this short timescale, advective transport and decay dominated, and stochastic perturbations have not yet accumulated sufficiently to separate individual trajectories. Beyond 6 h, however, particle dispersion grew steadily as particles experienced spatially heterogeneous velocity fields and random subgrid motions disrupted their coherence. After 24 h, the variance of particle positions increased, with greater variability between events reflecting both the cumulative effects of stochastic divergence and shifts in the center of mass (Figure 6a,c). However, at the quiet site, median dispersion remained below 1.25 km^2^ (Figure 6b) throughout the 24 h simulation, reaching only half the extent recorded at the dynamic site (2.5 km^2^). Similarly, inter‐event variability was very low for the quiet site compared to the dynamic site, as visible by the narrow band envelope (Figure 6a,b).

The no‐decay scenario illustrated the influence of advection and dispersion alone on particle density (Figure 6c,d). Both release sites displayed identical temporal patterns; they differed only in the magnitude of inter‐event variability. In the exponential and biphasic decay scenarios, the relative particle density (referenced to the median no‐decay results) declined smoothly and monotonically, with far less inter‐event scatter than observed for no‐decay (Figure 6c,d). Conversely, the delayed decay scenario produced an early surge: particle densities peaked at 2.5‐times those of the no‐decay scenario within the first 6 h, and then fell away in an approximately exponential decrease (Figure 6c,d).

Over the 24‐h period, the delayed and biphasic decay scenarios diverged significantly from the exponential decay in the early stages, with no overlap of percentile ranges for both sites, with the largest and longest lasting difference for the delayed decay scenario (Figure 6e,f). The delayed decay scenario exhibited an early surge in relative median density (normalized to the exponential reference), with the relative density reaching five times the exponential decay density by 6 h, reflecting early, rapid particle division, and stabilized afterwards, albeit with a gradually widening of the 20%–80% envelope indicative of growing between‐event variability. Thus, the tripling of the initial number of particles in the delayed decay scenario combined with the continuous removal of particles by decay in the exponential decay scenario led to a fivefold difference in densities after 6 h between the two scenarios. In contrast, the relative density in the biphasic scenario immediately plunged below the level of the exponential scenario to half its level, as rapid decay outpaced transport. After 19 h, the biphasic decay led to densities higher than the exponential decay, due to the decay slowdown during the second decay phase (Figure 6e,f).

## 4 Discussion

This study provides insights that both nuance and challenge the common exponential temperature‐dependent eDNA decay and its impact on eDNA dispersal in contrasting oceanic environments and the modeling of eDNA molecules. First, in our laboratory experiments, a second‐order model provided the best fit to the observed patterns of DNA concentration decline, with a delayed decay particularly visible at intermediate temperatures (13°C and 20°C compared to 27°C). This model captured the initial increase of eDNA copies followed by a decay phase, offering a more realistic description of degradation dynamics compared to the simpler one‐phase exponential model. Second, we incorporated these alternative decay patterns in Lagrangian particle tracking simulations, to the relative importance of the decay process in two contrasted sites. We showed that the decay process is a major driver of eDNA detectability at sites with weak or steady hydrodynamics, where eDNA plumes remained compact and traveled over modest distances, compared to sites characterized by fast currents and strong tidal forcing, where the greater variability between successive release events implies a lower probability of detection at any sampling location in the vicinity, and thus a lower relative influence of the decay process considered. Third, our modeling framework showed that tracking the weighted centroid of eDNA particles, rather than averaging over thousands of individual trajectories, provided a concise and robust representation of bulk eDNA motion.

### 4.1 Effect of Decay Process on the Dispersal of eDNA

Fine understanding of the decay process during the first hours appears crucial, since our modeling results demonstrated that the eDNA decay process type can exert a measurable influence on eDNA fate, with the decay scenario accounting for approximately 17% (main effect and interactions) of the total variance across all standardized output dispersal metrics for a highly dynamic environment with strong currents and high variability at the dynamic site, while up to 25% in the more homogeneous area (quiet site; Figure 4). Both the distances traveled and the spatial spread of particles were governed primarily by ambient currents and the prescribed diffusivity, yielding nearly identical mean displacements from the ensemble center of mass for all four decay scenarios (Figure 5a,b). Spatio‐temporal variability in transport was evident not only at seasonal scales, as reported by Zanni et al. (2025) and Pastor Rollan et al. (2024), but also on an hourly basis, reflecting the region's highly dynamic currents.

As expected, our results confirmed that, once eDNA has left the source, the decay formulations we tested had only a minor influence on horizontal spread; they only differ by their overall dispersal duration. Hydrodynamic forcing was the dominant control: the dynamic site experienced strong barotropic tidal currents; the higher velocities produced larger center of mass displacements and wider plume dispersion (Figures 2 and 6a). By contrast, the quiet site was located in a relatively calm area with only weak background currents. As a result, both dispersion and travel distance remained modest, and without the strong push‐and‐pull of tides the eDNA plume remained compact (Figures 2 and 6b). Taken together, these results suggest that in energetic settings (fast currents, pronounced tides) eDNA particles are diluted and dispersed rapidly, leading to greater inter‐event variability and reduced detectability, regardless of the modeled decay process. Conversely, in hydrodynamically steady regions eDNA remains more clustered, increasing the probability of detection at any given sampling location.

A meta‐analysis of nine field studies showed that mean horizontal eDNA travel is < 40 m in lakes but roughly 0.3–10 km in coastal seas, with 99% of particles retained within 0.5–20 km of their source (Jo et al. 2025). In our simulations, the daily median displacement of the particles' center of mass never exceeded 12 km, comfortably inside this empirically derived marine envelope. Independent modeling results also provided comparable values: depending on the value of the decay constant, mean particle transport distances ranged from 3.3 to 9.4 km (Zanni et al. 2025). Crucially, our results showed that in high‐current settings, the best window for eDNA sampling is the first 7 h after release and within roughly 9 km of the source. During this period, dispersion is still limited and the fragments remain tightly clustered around the center of mass (Figure 6a).

### 4.2 Decay Process Functional Form

In the literature review, experimental eDNA decay was found to be best described by an exponential function in around 36% of studies, while for the other 64%, a delayed or biphasic model seemed more appropriate. In our own experimental study, the model representing delayed decay also showed a better fit and was more capable of describing the strong initial increase in the number of DNA copies observed at 13°C and 20°C. At 27°C, this pattern was little observed, which might have been due to overall higher decay at this temperature (Figure 3). Thus, these results point to a temperature‐dependent smooth transition from monophasic to delayed decay. A delayed decay pattern has also been reported in several lotic eDNA (Nevers et al. 2020; Seymour et al. 2018; Snyder et al. 2023; Wood et al. 2020) and marine studies (Andruszkiewicz Allan et al. 2021). However, in many of the studies identified in our literature review, it was either ignored or attributed to improper sample mixing near the eDNA source (Brandão‐Dias et al. 2023); as of today, this may have hindered its importance.

As stated in the Introduction, environmental DNA may also sorb onto organic or inorganic particles, or become trapped in benthic biofilms; when these biofilms are later resuspended, they release the DNA back into the water column, creating non‐exponential decay patterns (Snyder et al. 2023).

In our experimental study, water samples were drawn from scientific seabass rearing tanks with a continuous water throughflow of sand filtered water from the nearby Bay of Brest which might have had reduced microbial activity. Advancing our understanding of eDNA decay will require a more detailed characterization of its intracellular and extracellular fractions (Nagler et al. 2022). For future studies, we recommend high‐frequency sampling during the first 0–12 h after eDNA release. Dense coverage of this early phase is essential for resolving the rapid decay dynamics and ultimately, for refining our understanding of the entire decay process.

### 4.3 Modeling Approach and Implications of Decay Process for eDNA Monitoring

In this study we monitored each simulated eDNA plume for the first 24 h after release, calculating hourly dispersal metrics for every decay scenario to track its complete temporal evolution. While Zanni et al. (2025) pooled trajectories by release event to explore multiple environmental drivers, here the objective was to isolate the effect of decay formulation alone. We therefore characterized particle transport by the displacement of the plume's center of mass. Following this single point rather than averaging thousands of individual particle paths from their release locations provided a concise and robust view of bulk motion, capturing how the entire eDNA cloud shifted through time while remaining insensitive to a few long‐range rare events. This center of mass approach, also used by Andruszkiewicz Allan et al. (2021), offers a physically intuitive, directly comparable measure for evaluating how alternative decay processes reshape eDNA dispersal over the first day after release. It also appears much more efficient to provide monitoring recommendations.

Although we did not directly model detection probabilities, the simulated density patterns revealed pronounced differences among decay scenarios (Figure 6c–f). Both sites exhibited the same median temporal evolution in density trajectories, while the main difference between the dynamic and the quiet sites was the spread of their density distributions: the dynamic site exhibited a much wider 20%–80% inter‐quantile range, indicating greater variability, whereas the quiet site's densities were far more tightly clustered (Figure 6c–f). Under the delayed decay scenario, particle density rose to nearly five times the exponential‐decay baseline within the first 6 h, suggesting a substantially extended detection window compared to both the simple exponential and the biphasic decay scenarios (Figure 6e,f). In contrast, the later decay scenario showed lower densities initially but, owing to its attenuated second decay phase, surpassed the exponential curve around 19 h post‐release indicating a late‐stage advantage for detecting eDNA (Figure 6e,f). The hypothetical *No‐decay* scenario, included to isolate advective and diffusive effects, showed continuous density accumulation and extreme variability between events for the quiet site (Figure 6e,f), underscoring how fragmentation and decay jointly regulated the spatial concentration of eDNA. Notably, the delayed decay scenario also exhibited greater event‐to‐event variance in density for the dynamic site: higher fragment counts amplified dispersion, widening the area over which concentrations fluctuated (Figure 6e,f).

Taken together, our results support growing empirical evidence that non‐monotonic, multi‐phase decay processes, that may occur in natural systems, might prolong and reshape the spatio‐temporal window of eDNA detection probabilities beyond the predictions of a single exponential model (e.g., Harrison et al. 2019; Andruszkiewicz Allan et al. 2021). For survey design, assuming simple first‐order decay may underestimate early‐hour detection success under delay dominated decay, yet overestimate late hour detection when a rapid first phase prevails. These results indicate that the decay process becomes most critical in weak‐current regions with relatively steady flow. At the quiet site, density curves showed little to no overlap (Figure 6f), whereas at the dynamic site substantial overlap persisted (Figure 6e). Consequently, in turbulent areas the likelihood of detecting eDNA will be higher, even without precise knowledge of the decay process, while in areas less dynamic, the scenario becomes critical to determining the relative density. In a theoretical “no‐current” simulation, density curves like those in Figure 6c–f would be expected to align almost perfectly, exhibiting virtually no variability aside from minimal diffusion.

## 5 Conclusions

One of the major knowledge gaps on delayed and biphasic decay is their occurrence and frequency in marine ecosystems. Our experimental results suggested that delayed decay might be more common at lower temperatures. Although it will present operational challenges and substantial cost, future field studies should prioritize the determination of the decay process in situ and under diverse conditions. To avoid misrepresenting biodiversity patterns or local differences, range shifts or abundance trends using eDNA‐based observations, this effort seems now crucial, in particular for eDNA‐based monitoring in environments characterized by steady or weak hydrodynamism. Ignoring the existence of decay processes that are not exponential may otherwise lead to bias in estimates of local diversity and abundance due to a complex relationship between shed eDNA and observable eDNA concentrations at different distances from the source.

## Author Contributions

**Mohamed Yosri. Zanni:** conceptualization (equal), formal analysis (lead), methodology (equal), writing – original draft (lead), writing – review and editing (equal). **Verena M. Trenkel:** conceptualization (equal), funding acquisition (equal), methodology (equal), writing – review and editing (equal). **Camille. Albouy:** resources (equal), writing – review and editing (equal). **Ana C. Vaz:** methodology (equal), writing – review and editing (equal). **Claire B. Paris:** methodology (equal), writing – review and editing (equal). **Robin Faillettaz:** conceptualization (equal), funding acquisition (equal), methodology (equal), writing – review and editing (equal).

## Funding

This work was supported by Conseil Régional des Pays de la Loire, Institut Français de Recherche pour l'Exploitation de la Mer, Agence Nationale de la Recherche (Grant ANR‐24‐CE91‐0019).

## Ethics Statement

The authors have nothing to report.

## Conflicts of Interest

The authors declare no conflicts of interest.

## Supporting information


**Data S1:** ece372987‐sup‐0001‐supinfo.docx.

## Data Availability

This study was conducted using MARS3D hydrodynamic data (https://doi.org/10.12770/3edee80f‐5a3e‐42f4‐9427‐9684073c87f5). The Connectivity Modeling System (CMS) source code is openly available at https://github.com/beatrixparis/connectivity‐modeling‐system. Experimental data are available at https://doi.org/10.17882/111489.
