# Supplementary material for: Beyond Exponential Decay: How Biphasic and Delayed Decay Dynamics Shape Marine eDNA Dispersal
Source: Ecol Evol. 2026 Jan 25;16(1):e72987. doi: 10.1002/ece3.72987 (PMC12832204; doi:10.1002/ece3.72987)
Supplement: Supplementary file 1 — Data S1: ece372987‐sup‐0001‐supinfo.docx. [file ECE3-16-e72987-s001.docx]

**Supporting Information for:**

**Beyond Exponential Decay: How Biphasic and Delayed Decay Dynamics Shape Marine eDNA Dispersal**

***Table S1.*** *Summary of information extracted from the reviewed studies reporting eDNA decay dynamics. Process descriptions are based on visual inspection of time series plots of eDNA/RNA concentrations.*

| Reference | Decay process category | Process description | Decay rate estimated in the study | Methods | Sampling times | Sampling in first 12 h | Type | Species | Description | Temperature |
| --- | --- | --- | --- | --- | --- | --- | --- | --- | --- | --- |
| Shogren et al. 2018 | Biphasic | Rapid initial loss of eDNA within the first hours, followed by a slower secondary phase. | Primary decay constants (k₁) ranged from ~5–35 d⁻¹ depending on fragment size and treatments; secondary constants (k₂) were consistently lower. Breakpoints (t′) occurred within 4–24 h. | 15 artificial recirculating stream flumes (40 L each) seeded with rainbow trout (Oncorhynchus mykiss) eDNA. Treatments included three flow velocities (0.1, 0.5, 0.85 m s⁻¹) and five levels of substrate biofilm cover (0–100%). | 250 mL water samples collected frequently over 10 days (15 min to daily intervals). eDNA quantified with a nested primer assay targeting fragments of 97, 455, and 697 bp. | Yes | DNA | Rainbow trout eDNA (from hatchery fry water) | eDNA consistently followed biphasic exponential decay, with biofilm cover accelerating initial removal (higher k₁) and fragment size strongly influencing persistence (short fragments persisted longest). Flow velocity had little direct effect on decay constants but alters transport distances. | 14-18 °C |
| Trafford et al. 2024 | Delayed | eDNA increased (~×1.5) to a maximum between ~4–12 h (replicate-specific), then declined. | T99 = 18.9–52.4 h ⇒ k ≈ 0.088–0.244 h⁻¹ overall; Bd examples k = 0.124–0.243 h⁻¹ across 15/20/25 °C. | 2-L glass microcosms with sterile pond water; inoculated with Bd zoospore suspension or ranavirus lysate; incubated at 10, 15, 20 °C ± light; qPCR targeting ITS-1 (Bd) and MCP gene (ranavirus) | 2, 4, 8, 12, 24, 48, 72, 96, 120, 144, 360, 672 h (up to 28 days). | Yes | DNA | Chytrid fungus (*Batrachochytrium dendrobatidis*) and ranavirus | Estimated constant k for each condition; compared decay rates under different temperatures and photoperiods | 15, 20, 25 °C |
| Dercksen et al. 2025 | Delayed | In the high-flow treatment, concentrations exhibited a transient peak (~48 h; ~x2–x3) followed by single-phase exponential | no-flow k ≈ 0.0033 h⁻¹ (t½ ≈ 210 h); low-flow k ≈ ~0 (CI includes 0); medium-flow k ≈ 0.0023 h⁻¹; high-flow k ≈ 0.0049 h⁻¹ after the peak. | Closed 700 L annular flume; one-off pulse of ~60 L zebrafish rearing water; top-lid rotation set to 0 / 0.35 / 1.05 / 1.80 m s⁻¹ (no, low, medium, high). | 0, 3, 6, 12, 24, 48, 72, 96, 144, 168 h | Yes | DNA | Zebrafish (*Danio rerio*) | Assessed how flow velocity affects eDNA persistence and detectability; fitted an exponential model to each time-series | 15.3 ± 1.1 °C |
| Barnes et al. 2014 | Exponential | Single-phase exponential decay of Common Carp eDNA in lab mesocosms | k = 0.105 h⁻¹ | 21 aerated 20 L mesocosms; treatments were 0, 1, or 3 goldfish per mesocosm to create gradients in BOD, chlorophyll a, total DNA, pH (used as an environmental index). Example day-7 values: BOD (mg/L) 1.6 / 4.8 / 9.4; Chl a (µg/L) 2.3 / 257.0 / 484.5; Total DNA (µg/L) 7.7 / 96.8 / 276.4. | 8, 16, 24, 72, 96, 120, 168, 336, 504 h | Yes | DNA | Common Carp (*Cyprinus carpio*) eDNA (goldfish used only to manipulate water quality). | Test how environmental conditions (BOD, chlorophyll, pH, total DNA) influence eDNA decay and detectability. | 25 °C |
| Bylemans et al. 2018 | Biphasic | Weibull fits show fast→slow (β<1) | Exponential k ≈ 0.11–0.77 day⁻¹ (≈ 0.0046–0.032 h⁻¹); implied t½ ≈ 0.9–6.3 days (≈ 22–151 h | Indoor tanks at 20 °C, 12 h light; HD/MD/LD densities (1 fish per 10/30/60 L); UV-sterilized tap water, gravel, aeration; 50 mL samples filtered on 1.2 µm glass fiber; SYBR qPCR targeting COI 96/285/515 bp (mtDNA) and ITS 95 bp (nuDNA). | While fish present 0, 2, 6, 24, 72, 168, 336 h; after removal 338, 342, 360, 408, 504, 672, 1008 h. | Yes | DNA | Common Goldfish (Carassius auratus) | Test decay vs fragment length/origin; compare exponential vs Weibull. | 20 °C |
| Brandão-Dias et al. 2023 | Delayed | Lag ~6 h, ~×2 eDNA peak then decline; decay differs by particle size | Aquatic mesocosms combining carp (moat water) and rainbow trout (hatchery tank water); sequential filtration at 10 µm / 1.0 µm / 0.4 µm / 0.2 µm; ddPCR targeting mitochondrial Cytb for both species. Prefiltering (10 or 1 µm) was used to test replenishment/cascading from larger to smaller sizes. | Three filter cut-offs tested for size-dependent decay | 1, 6, 18, 30 h | Yes | DNA | Common Carp & Rainbow Trout | Sequential filtration revealed particle-size-dependent eDNA decay | 20 °C |
| Nevers et al. 2018 | Delayed | 0–6 h increase then decay | Round Goby eDNA; k ≈ 0.043–0.058 h⁻¹ at 12–19 °C | Indoor mesocosms (9.5 L glass aquaria with 7 L “source” water), controlled chamber at 19 °C or 12 °C; qPCR COI assay; mesocosm shedding phase then fish removed for decay phase; plus Lake Michigan/Huron field validation. | C (19 °C): 0, 6, 24, 48, 72, 168, 216, 312 h; E (12 °C): 0, 6, 24, 48, 72, 96, 144, 216, 312, 360 h. | Yes | DNA | Round Goby (Neogobius melanostomus) | eDNA used to quantify an elusive invasive fish | 12 & 19 °C |
| Scriver et al. 2024 | Fluctuating | Non-monotonic eDNA/eRNA peaks driven by microbes | No clear exponential rate; eRNA degrades faster than eDNA | Dialysis-membrane diffusion system, natural vs chilled seawater | 0, 3, 6, 9, 12 h | Yes | DNA & RNA | Marine microbial community (metabarcoding) | Unexpected re-increases of eDNA/eRNA; chilled water more stable | Ambient vs 4 °C (chilled) |
| This study 2021 | Delayed | Lag ~6 h, ~×3 peak then decay | k = 0.05 h⁻¹ @13 °C; 0.04 @20 °C; 0.03 @27 °C | 120 L aquaculture water → 2×20 L tanks ×3 T; ddPCR, 7 timepoints | 0, 4, 6, 10, 16, 22, 26.5 h | Yes | DNA | European Sea Bass (Dicentrarchus labrax) | Observed lag & peak; two replicates per temperature | 13, 20, 27 °C |
| Wood et al. 2020 | Exponential | Exponential decay (RNA) | k = 0.682, 0.182, 0.156, 0.182 | 15 L aquaria, 50 mL samples; 4 h pre-control | 4, 8, 12, 24, 72, 120, 168, 336, 504 h | Yes | RNA | Sabella spallanzanii & Styela clava | Settling/binding to surfaces; ephemeral design | 13 °C |
| Wood et al. 2020 | Exponential | Exponential decay (DNA) | Isolated vs combined k: 0.248 / 0.338 vs 0.104 / 0.125 | Same tanks as RNA experiment, DNA focus | 4, 8, 12, 24, 72, 120, 168, 336, 504 h | Yes | DNA | Sabella spallanzanii & Styela clava | Compared isolated vs combined treatments | 13 °C |
| Jo et al. 2021 | Biphasic | all targets in tanks show an initial rapid loss (k₁) followed by a slower phase (k₂). | k₁ and k₂ estimated per target fragment with CIs; no single global k. | From a 200-L stock tank (5 jack mackerel), two 60-L sampling tanks were filled; BAC 0.01% added to one. 1 L samples filtered on GF/F 0.7 µm; TaqMan qPCR for mt CytB (164, 682 bp) and nu ITS1 (164, 603 bp). | Tanks: 0, 6, 12, 24, 48, 72, 96 h. Field: 0, 6, 24 h. | Yes | DNA | Japanese Jack Mackerel | Tests BAC preservation efficacy across targets/fragment lengths; shows BAC mainly suppresses microbially driven fast phase and maintains species richness in metabarcoding through the day. | 18 ± 1 °C |
| Jo et al. 2023 | Biphasic | Rapid→slow decay (10–20 % retained) | k₁,k₂ vary by T & biomass | 200-L tanks, four temperatures (13/18/23/28 °C), three biomass levels (small/medium/large); fish removed, then decay tracked; filtration 0.7 µm GF; qPCR mt cyt b & nu ITS1; | 0, 2, 4, 8, 16, 24, 48, 72, 96 h | Yes | DNA | Japanese Jack Mackerel | Quantifies how temperature, biomass, and marker type affect k₁ vs k₂ in a biphasic framework. | 13–28 °C |
| McCartin et al. 2022 | Biphasic | Rapid initial loss, then a slower phase. Initial phase not controlled by T/pH/DO; second phase strongly temperature-controlled, pH weak, DO not significant. | Initial k ≈ 0.07–0.084 h⁻¹ (4–20 °C; no significant predictors); second-phase k ≈ 0.011 h⁻¹ (10 °C) to 0.036 h⁻¹ (20 °C). | 42 L artificial seawater (35 PSU) in opaque carboys; 11 combos of T = 4/10/20 °C, pH = 7.6/7.9/8.2, DO = 0.1/3.6/7.2 mg L⁻¹; source eDNA from Lophelia tissue homogenate; 0.22 µm Sterivex; qPCR on 154 bp COI. | 0, 1, 2, 4, 8, 24, 48, 72 h and weekly to 2 w | Yes | DNA | Lophelia pertusa | Demonstrates >2 weeks persistence at ≤10 °C vs ≤1 week at ≥20 °C; proposes a temperature-dependent persistence model. | 4–20 °C |
| Andruszkiewicz Allan 2021 | Delayed | For both jellyfish (moon jelly *Aurelia aurita* and sea nettle *Chrysaora* spp.), eDNA concentrations increase from 0 to 6 h across several temperature treatments, before declining thereafter | Rates vary by species & temperature | Three dark temperatures (6, 15, 23 °C) plus a room-temperature (~20 °C) ambient-light treatment; duplicate 400 mL samples; 0.22 µm Sterivex; qPCR. | 4× in first 24 h then daily to 17–100 d | Yes | DNA | Mummichog (*Fundulus heteroclitus*), grass shrimp (*Palaemon* spp.), moon jelly (*Aurelia aurita*), sea nettle (*Chrysaora* spp.) | Decay rates vary by species and temperature: mummichog and grass shrimp show faster decay at higher T; scyphozoan jellies show no clear T-dependence. | 6, 15, 23 °C (+ ~20 °C ambient-light). |
| Eichmiller et al. 2016 | Biphasic | Biphasic in the trophic-state experiment | In the trophic-state experiment, k₁ ranged ~0.66–2.34 d⁻¹ with k₂ ≈ 0.02–0.43 d⁻¹ depending on water type | eDNA was seeded by holding 10 carp in 30 L lake water for 1–2 h, then incubated as 50 mL glass microcosms (dark). | Pre, 0, 0.25, 0.5, 0.75, 1, 2, 3, 5, 7, 10, 14, 21, 28 days | Yes | DNA | Common Carp (Cyprinus carpio) | Fastest decay in oligotrophic water;dystrophic/well slowest; decay increased with temperature. | 5, 15, 25, 35 °C |
| Collins et al. 2018 | Delayed | Initial increase (~0–12 h) in summer for the mixed-offshore treatment (Offshore two-thirds)—and occasionally Inshore two-thirds | k ≈ 0.015–0.033 h⁻¹ depending on treatment/species (t½ ≈ 21–46 h) | 24 aquaria (9 L) with five water treatments (offshore, two mixed ratios, inshore, plus synthetic control); tanks spiked with eDNA-rich water from shanny and shore crab stocks; 0.22 µm Sterivex; qPCR COI. | 0, 12, 24, 48, 96, 192 h | Yes | DNA | Shanny (*Lipophrys pholis*), Shore crab (*Carcinus maenas*) | Salinity & origin affected early rise | Aquarium room at 10 °C (winter) and 15 °C (summer) |
| Sassoubre et al. 2016 | Biphasic | Rapid then slower removal; ~10–20 % remains | k₁,k₂ vary by species | One ~5200 L seawater mesocosm; four runs (anchovy only; sardine only; mackerel only; mackerel+sardine). 0.2 µm filters; TaqMan qPCR (genus-specific). | Sampling: 3–8× per day with fish in tank; after removal, 2–3× per day for 3–4 days | Yes | DNA | Northern anchovy, Pacific sardine, Pacific chub mackerel. | Shedding and decay quantified in large tank | 15 °C |
| Kutti et al. 2020 | Delayed | Early 0–5 h increase ×2–3 then decay | k = 0.017 h⁻¹ (t½ ≈ 41 h) at 8 °C. | 0 L microcosms (8 L) at 8 °C; Lophelia pertusa colonies; sampling at 0, 5, 10, 24, 48, 96, 168 h; filtration 0.45 µm; ddPCR (178 bp mtDNA control region). | 0, 5, 10, 24, 48, 96, 168 h | Yes | DNA | Lophelia pertusa (syn. *Desmophyllum pertusum*). | Lab decay quantified; field mapping + Lagrangian dispersion modeling (LADIM/ROMS) used eDNA as passive particles to predict reef signals. | 8 °C |
| Snyder et al. 2023 | Delayed | early 6 h uptick observed only in open (light) mesocosms with no substrate (“breakout phase”)(×4) | Total eDNA k ≈ 10.2 d⁻¹ (biofilm) vs 2.0 d⁻¹ (bare) vs 1.1 d⁻¹ (no substrate); by size (bare): >10 µm = 15.7 d⁻¹, 1.0 µm = 3.9 d⁻¹, 0.2 µm = 2.9 d⁻¹. | 24 recirculating fiberglass mesocosms; treatments = open vs shaded and no substrate / bare / biofilm; sequential filtration 10–1.0–0.2 µm; ddPCR (carp cyt b, 78 bp). | 0.67, 6, 18, 48 h | Yes | DNA | Common Carp (Cyprinus carpio) | Biofilm strongly increases removal; larger particles (>10 µm) are removed faster than small (0.2–1.0 µm). | 20 °C (light vs dark) |
| Holman et al. 2022 | Delayed | Some species show ×3 increase then rapid loss | Species-specific k ≈ 0.0096–0.0348 h⁻¹ | 50-L aquarium at 13 °C (dark/covered) assembled from a rocky-shore community; water transferred to experimental tanks; 0.22 µm Sterivex; COI (313 bp) | 1, 3, 5, 18, 24, 48, 72, 120, 192 h | Yes | DNA (metabarcoding) | Marine metazoan community | Most stocked species became undetectable after ~48 h”; decay constants estimated per species | 13 °C |
| Xue Yu et al. 2022 | Delayed | 2–3-day increase then decay (T-dependent) | Exponential fits (days 3–14) give average daily loss ≈ 25.5 % at 20 °C, 31.6 % at 20 °C+UV, and ~2.9 % at 4 °C | 5 L lake water in sterile glassware; 0.45 µm filtration; qPCR of 18S (eukaryotes) and 16S (prokaryotes); UV set to 10 µW cm⁻² in the 20 °C+UV treatment. | 0, 1, 2, 3, 4, 5, 7, 9, 11, 14 d | No | DNA | NA(community signal) | An early rise (<3 d) likely from residual tissues/algae, then exponential decay; UV had little additional effect relative to temperature. | 4 vs 20 °C |
| Andruszkiewicz et al. 2017 | Exponential | Exponential (first-order) | k = 0.039 h⁻¹ (surface) and 0.038 h⁻¹ (depth) → t½ ≈ 18 h. | Dialysis bags (500 mL) in a ~10,000 L outdoor seawater tank, suspended 5 cm vs 70 cm below surface; 0.22 µm filters; qPCR for *Scomber japonicus* (COI) + 12S metabarcoding. | 0, ~14.8, 24.5, 38.7, 48.3, 63.0, 72.8, 87.0 h (≈ every 12 h over 4 days) | No | DNA | target qPCR = *Scomber japonicus*; community fish by metabarcoding | Surface vs depth (more vs less UV). No significant difference between treatments → sunlight is not the main driver. | ~16.8 °C, |
| Minamoto et al. 2017 | Exponential | Exponential or near-exponential | k = 0.0329 h⁻¹ ⇒ t½ ≈ 21.1 h. | 3 × 500 L flow-through seawater tanks; 1 L samples; GF/F 0.7 µm filtration; TaqMan qPCR (COI). | 1, 2, 4, 8, 16, 24, 48, 72, 120, 168 h | Yes | DNA | Japanese sea nettle (*Chrysaora pacifica*) | eDNA reflects jellyfish distribution | ~17–20 °C |
| Kwong et al. 2021 | Exponential | Exponential (decay) | k = 0.034, 0.050, 0.068 h⁻¹ at 24/26/28 °C | 500 mL bottles incubated at 24/26/28 °C; 1.2 µm cellulose nitrate filtration; ddPCR COI. | 0, 3, 6, 12, 24, 48, 72, 96, 120 h | Yes | DNA | Acanthaster cf. solaris | Shedding & decay across temperatures | 24/26/28 °C |
| Kirtane et al. 2021 | Delayed | Post-removal, a 1–3 h uptick above t=0 (x 1–5) is visible for black sea bass summer flounder (both systems ) | k ≈ 0.07–0.57 h⁻¹ depending on species/system (t½ ≈ 1.2–9.9 h) | Two replicate recirculating aquaculture systems (RAS) with seawater; fish added to reach steady state, then removed for decay phase; 0.4 µm filters; species-specific TaqMan qPCR (COI). Field bottom trawls + paired eDNA were also done for comparison (both mostly non-detect/low catch). | (BSB/WF decay) 1, 3, 6.5, 9, 12, 15, 18, 21, 24, 27, 30, 33.5, 37, 41, 46, 51, 60, 84, 120 h; (SF decay) 2, 9.5, 12.5, 15.5, 24.5, 36.5, 46, 62, 85, 97, 109, 120, 132 h. ≤12 h: yes | Yes | DNA | Black sea bass (*Centropristis striata*), Winter flounder (*Pseudopleuronectes americanus*) (135 & 292 bp assays), Summer flounder (*Paralichthys dentatus*) | Quantified shedding (pre-removal) and decay (post-removal); compared with trawl surveys (agreement at low abundance). | RAS means ≈ 19.9 ± 1.5 °C (BSB/WF runs) and 16.2 ± 0.1 °C (SF runs) |
| Jo et al. 2017 | Exponential | Single-phase exponential decay | k = 0.044 h⁻¹ (t½ ≈ 15.8 h) and k = 0.090 h⁻¹ (t½ ≈ 7.7 h) | Rearing water from 3×200-L tanks (jack mackerel) was isolated to sampling tanks; 1 L samples; GF/F 0.7 µm; TaqMan qPCR (CytB, 127 & 719 bp). | t=0, then 0.5, 1, 1.5, 2, 4, 6, 8, 10, 12, 14, 16, 18, 20, 22, 24, 28, 32, 36, 40, 44, 48 h | Yes | DNA | Japanese jack mackerel (*Trachurus japonicus*) | Two amplicon lengths compared | 25–29 °C |
| Sigsgaard et al. 2016 | Exponential | Sun vs shade buckets; exponential decay over days | Not reported | Two 90 L outdoor buckets (sun vs shade) from a 6×30 L composite; 0.22 µm Sterivex; qPCR | ~every 12 h for 8 d (more frequent first 3 d) | Yes | DNA | Whale Shark (Rhincodon typus) | authors fitted an exponential decay to sun vs shade buckets but did not report a numeric k or half-life in the main text. | 29–43 °C, |
| Thomsen et al. 2012 | Exponential | Single-phase exponential decay | k = 0.0134 h⁻¹ (t½ ≈ 51.7 h) and k = 0.0292 h⁻¹ (t½ ≈ 23.7 h). | 50 L seawater in a 54 L aquarium, 15 °C, 12:12 h light, circulation; species-specific TaqMan qPCR (cytb, 101–104 bp); 400 mL subsamples; 8 qPCR reps/sample. | t≈0 (within 1 h) then frequent “hours→days” over ~15 days (total 19 samples) | Yes | DNA | European flounder (*Platichthys flesus*), three-spined stickleback (*Gasterosteus aculeatus*). | First marine eDNA metabarcoding test of local fish biodiversity from seawater, benchmarked against 9 conventional survey methods, plus a controlled aquarium experiment quantifying first-order eDNA decay for two fish species at 15 °C. | 15 °C |
| Cowart et al. 2018 | Exponential | Exponential (first-order) | k = 0.234 day⁻¹ (≈ 0.0098 h⁻¹); t½ = 37.2 h. | Two 20 L carboys filled from a 2,000 L flow-through tank housing *Chionodraco rastrospinosus*; carboys submerged in the tank; 1 L/day filtered on 0.45 µm nylon; qPCR targeting ND2 (70 bp). | Daily for 20 days (no sub-24 h timepoints). | No | DNA | Antarctic icefish (*Chionodraco rastrospinosus*). | Metagenomic eDNA survey of WAP + controlled decay of icefish ND2 to estimate persistence in polar waters. | −1.04 ± 0.12 °C (carboys held in ambient flow-through) |
| Forsström & Vasemagi 2016 | Exponential | After removing crabs (day 8), eDNA declined exponentially over 7 d | k ≈ 0.0546 h⁻¹, t½ ≈ 12.7 h | Aquarium microcosms (not mesocosms): 10 L plastic tanks, 5.6 psu, 17 °C, 10L:14D; four male *R. harrisii* (one per tank). 15 mL samples preserved with NaOAc/ethanol; qPCR (TaqMan) COI, 75 bp.I | Days 1, 3, 5, 8; post-removal decay days 1, 2, 3, 5, 7. | No | DNA | Mud crab (Rhithropanopeus harrisii) | eDNA decay in brackish conditions | 17 °C |
| Jo et al. 2020 | Exponential | Extended first-order incl. T & biomass | nu-eDNA: *t*½ ≈ 1–5 h (k ≈ 0.143–0.697 h⁻¹)  mt-eDNA: *t*½ ≈ 1.5–19 h (k ≈ 0.0366–0.469 h⁻¹) | 200-L tanks; qPCR ITS1 & CytB; 0–96 h. | 0, 2, 4, 8, 16, 24, 48, 72, 96 h | Yes | DNA | Japanese Jack Mackerel | Quantify eDNA shedding and decay rates for Japanese jack mackerel and test how they depend on water temperature and fish biomass | 13–28 °C |
| Saito & Doi 2021 | Biphasic | (Biphasic) for pond Sterivex cells & carp | Sterivex — IPC sea 0.024, IPC pond 0.279, Okis sea 0.181, Okis pond 0.171, Carp pond 0.109, Jack mackerel sea 0.112. Filtrate — IPC sea 0.509, IPC pond 0.439, Okis sea 0.151, Okis pond 0.082, Carp pond 0.022. → t½ ≈ 1.4–31.5 h across targets/fractions. | Bottle experiments (not mesocosms): seawater (Seto Inland Sea) and pond water, 500 mL per sample on 0.45 µm Sterivex + 1.5 mL filtrate; qPCR for IPC, coho cells (Oncorhynchus kisutch), carp (pond), jack mackerel (sea) | 0, 0.5, 1, 3, 6, 12, 18, 24, 48, 72, 120, 168 h | Yes | DNA | C. carpio (pond), T. japonicus (sea), O. kisutch cells; IPC fragment DNA. | Comparative tests in different waters & artificial gene controls | ~25 °C |
| Qian et al. 2022 | Exponential | eDNA shows strong temperature-dependent exponential decay; eRNA also exponential but less temperature-sensitive. | eDNA k = 0.011–0.486 h⁻¹ → t½ ≈ 63–1.4 h (10→25 °C); eRNA k = 0.190–0.379 h⁻¹→ t½ ≈ 3.6–1.8 h. | Post-removal tank water split into 4 × 10 L tanks at 10/15/20/25 °C; 3 µm filtration; qPCR COI (106 bp) for eDNA and eRNA (via cDNA). I | 0, 4, 8, 12, 24, 72, 120, 168, 336, 504 h | Yes | DNA RNA | Chinese prawns (Fenneropenaeus chinensis) | Quantifies temperature effects on eDNA vs eRNA decay; eDNA much more temperature-sensitive than eRNA. | 10–25 °C |
| Ellis et al. 2022 | Exponential | Rapid exponential decay; rates not significantly different between species. | k = 0.035 h⁻¹ (U. pinnatifida) / 0.041 h⁻¹ (A. amurensis) → t½ = 19.8 / 16.9 h | 3 × 20 L seawater containers at 15 °C (sealed, aerated); 0.22 µm Sterivex; species-specific TaqMan qPCR (assays from Bott 2010 / Bax 2006) | 0, 12, 18, 24, 36, 48, 72, 120 h | Yes | DNA | Kelp (*Undaria pinnatifida*); Northern Pacific seastar (*Asterias amurensis*) |  | 15 °C |
| Weltz et al. 2017 | Exponential | Exponential decay during cold storage at 4 °C; two DO levels (55% vs 20%) | Reported as β = 0.020 and 0.011 (interpretable as h⁻¹ from the time axis), giving t½ ≈ 34.7 h and 63 h. | Water taken from on-shore holding tanks that had housed skates; then 4 °C storage, subsampled every 24 h for 72 h (no sub-12 h points). Target marker mtDNA NADH4 qPCR. | 0, 24, 48, 72 h. | No | DNA | Maugean skate (*Zearaja maugeana*) | Tested how cold storage and dissolved oxygen affect short-term eDNA persistence. | 4 °C |
| Ely et al. 2021 | Delayed | Detection index metric over distance & time | Max detection ≈ 7.5 h | MiFish 12S metabarcoding; 1 L samples on 0.22 µm Sterivex. Dock transect | Dock transect sampling: every 1.5 h for the first 12 h, then 24, 48, 96 h. (Two additional transects for native community at 6, 12, 24, 48, 120 h.) | Yes | DNA | Grass Carp (Ctenopharyngodon idella) | In-situ persistence/transport test showing rapid dispersion and short | ambient |
| Murakami et al. 2019 | No direct 1st-order model | Distance-based in-situ decay | 0–36.5 % / h decline | Caged striped jack at pier; stations at 1, 10, 30, 100, 300, 600, 1000 m along two transects; 1 L surface samples; GF/F 0.7 µm; TaqMan qPCR (cytb, 130 bp). | After deployment 0, 1, 2, 4, 8, 24, 48 h; after removal 0, 1, 2, 4, 8, 24 h. Sampling | Yes | DNA | Striped Jack (Pseudocaranx dentex) | Field test of spatiotemporal dispersion & degradation; concentration decreased with distance (power regressions up to R²=0.93); vertical checks showed surface > mid/bottom at 37 h. | 21.4 ± 0.4 °C |
| Sheehan et al. 2024 | Exponential | Single-phase exponential decay | k = 0.154 h⁻¹; t½ = 4.5 h | Eight glass aquaria (30 L), aerated & covered; 0.45 µm MCE filters; qPCR COI (148 bp). Inoculum prepared from freeze-killed mysids; two water treatments (unfiltered vs 60 µm). | T₀ immediately, then daily ×5 days, then every 2 days ×6 days, then less frequent to day 29. | No | DNA | *Hemimysis anomala* (invasive mysid) | Quantifies zooplankton eDNA persistence in harbor-water microcosms to inform AIS monitoring in Great Lakes ports. | 19–23 °C |
| Nevers et al. 2020 | Delayed | 0–14 h increase then very fast decrease | Not reported | Stream with caged round goby; stations at 1–120 m; simultaneous high-frequency sampling during the first ~24 h, then 2, 4, 24 h; after cage removal, sampled again at 48 h. qPCR COI (130 bp); 0.45 µm filters. Lab: eight ~38 L tanks with sand (WB/PL), water & sediment sampled through 14 d (shedding) + 150 d post-removal (decay). | 0,1,2,4,6,12,24 h / River: 0,6,12,24,48,72 h | Yes | DNA | Round Goby (*Neogobius melanostomus*) | Quantifies shedding/decay in water vs sediment and downstream transport; shows most detections within ≤30 m, but models imply potential long-distance transport under some flows. | ~21.4 ± 0.4 °C |
| Seymour et al. 2018 | Exponential | Rapid monotonic decline | No k; authors report proportional loss per hour early in the run (e.g., hour-1 loss ~0.98 at acidic site vs ~0.60–0.67 at neutral). | Four 20-m recirculating flumes spanning pH ~5.3–6.8; bolus addition of eDNA-rich water from Daphnia magna, Ephemera danica, Anguilla anguilla; 1 L water samples, 0.22 µm Sterivex, species-specific qPCR; parallel biofilm scrapes. | −1, 0, 1, 3, 7, 19, 29, 43 h | Yes | DNA | *D. magna*, *E. danica*, *A. anguilla*. | Tests abiotic controls (especially pH) on lotic multi-species eDNA decay; shows faster loss at lower pH and limited biofilm accumulation. | ~14.5–16.2 °C |
| Che‑Pelicier et al. 2025 | Exponential | Single‑phase exponential loss of eDNA and eRNA; λ 0.01–0.23 h⁻¹ (eDNA) & 0.01–0.51 h⁻¹ (eRNA); faster at 30 °C, slower at 4 °C | eDNA λ = 0.012–0.123 → t½ = 7.5–57.8 h (across 4–30 °C, species & treatments)  • eRNA λ = 0.016–0.144 → t½ = 4.8–43.3 h | 12 wild eels (6 LF, 6 SF) held 30 h in 50 L freshwater aquaria (15 °C); single‑ & dual‑species treatments; water (100 mL) filtered (0.22 µm); eDNA/eRNA co‑extracted; ddPCR; subsamples incubated at 4 °C & 30 °C | 0, 4, 8, 12, 18, 24, 72, 120, 168 h | Yes | DNA & RNA | Longfin eel (Anguilla dieffenbachii); Shortfin eel (A. australis) | Compared release and decay of eDNA vs eRNA; eRNA consistently decayed ≈2× faster; temperature strongly modulated rates | 15 °C main; 4 °C & 30 °C subsamples |


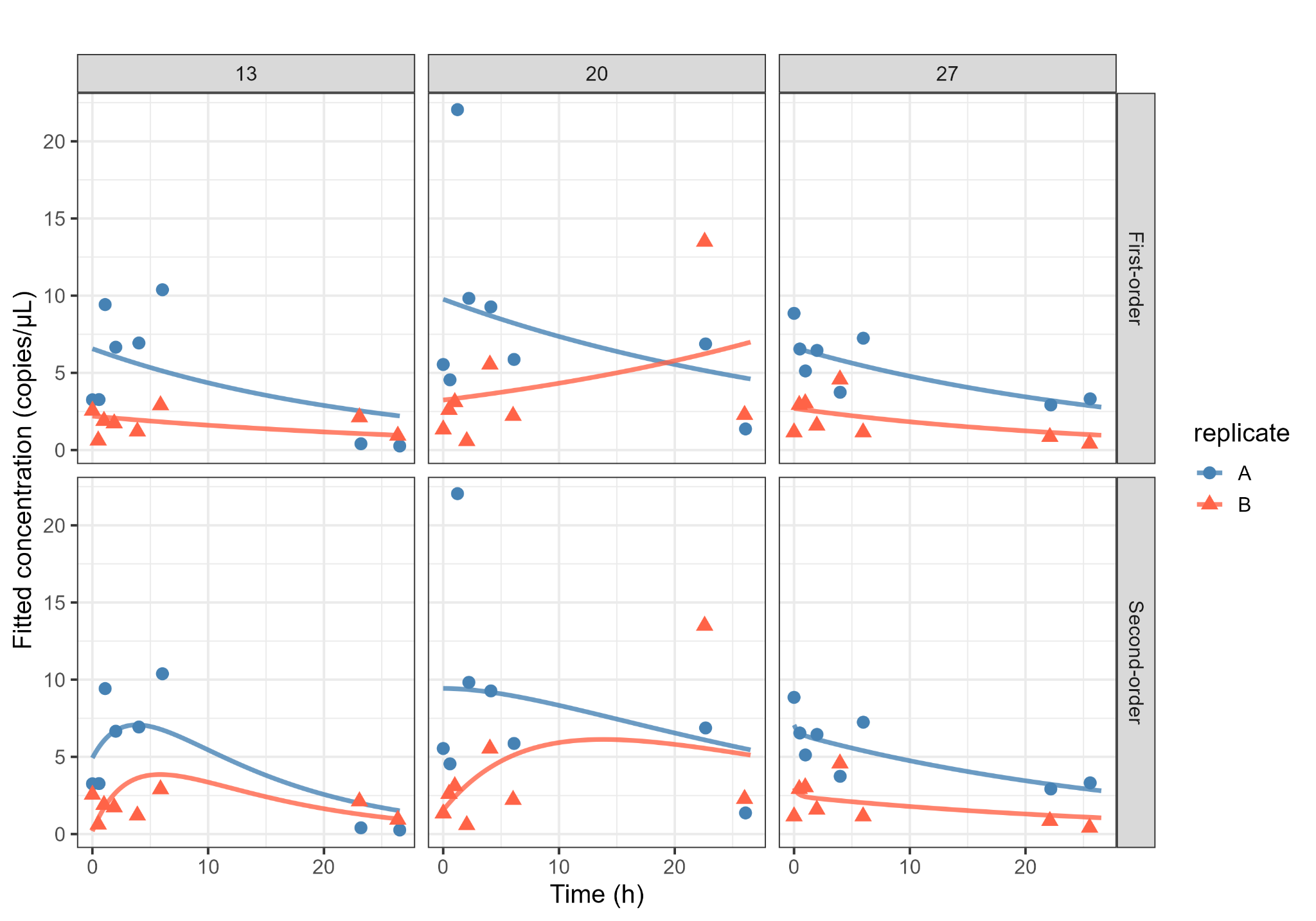


***Figure S1: Data (dots and triangles) and fitted first and second-order models (solid lines) for each experimental temperature and experimental replicate.***

***Table S2*** *Summary of fixed‑effect parameter estimates and performance metrics
from the nonlinear mixed‑effects decay models fitted to the experimental data.*

| **Model** | **C0**  **_(13°C)_** | **C0**  **_(20°C)_** | **C0**  **_(27°C)_** | **K**  **_(13°C)_** | **K**  **_(20°C)_** | **K**  **_(27°C)_** | **C0_1_**  **_(13°C)_** | **C0_1_**  **_(20°C)_** | **C0_1_**  **_(27°C)_** | **K_1_**  **_(13°C)_** | **K_1_**  **_(20°C)_** | **K_1_**  **_(27°C)_** | **C0_2_**  **_(13°C)_** | **C0_2_**  **_(20°C)_** | **C0_2_**  **_(27°C)_** | **K_2 (13°C)_** | **K_2 (20°C)_** | **K_2 (27°C)_** | **AIC** | **R^2^** |
| --- | --- | --- | --- | --- | --- | --- | --- | --- | --- | --- | --- | --- | --- | --- | --- | --- | --- | --- | --- | --- |
| First-order  (All Points) | 4,38 | 6,5 | 4,67 | 0,04 | 0 | 0,04 | NA | NA | NA | NA | NA | NA | NA | NA | NA | NA | NA | NA | 772,74 | 0,4 |
| Second-order  (All Points) | NA | NA | NA | NA | NA | NA | -8,36 | 12,75 | 0,53 | 0,3 | 0,03 | 2,77 | 10,92 | -7,26 | 4,5 | 0,08 | 0,12 | 0,03 | 771,67 | 0,45 |
| First-order (Excluding 22h timepoint) | 4,41 | 6,66 | 4,66 | 0,04 | 0,05 | 0,03 | NA | NA | NA | NA | NA | NA | NA | NA | NA | NA | NA | NA | 709,31 | 0,48 |
| Second-order (Excluding 22h timepoint) | NA | NA | NA | NA | NA | NA | -8,45 | -6,96 | -1,07 | 0,3 | 1,6 | -0,02 | 11,02 | 9,87 | 5,72 | 0,08 | 0,1 | 0,02 | 694,73 | 0,59 |

**References**

Andruszkiewicz Allan, E., Zhang, W.G., C. Lavery, A., F. Govindarajan, A., 2021. Environmental DNA shedding and decay rates from diverse animal forms and thermal regimes. Environmental DNA 3, 492–514. https://doi.org/10.1002/edn3.141

Andruszkiewicz, E.A., Sassoubre, L.M., Boehm, A.B., 2017. Persistence of marine fish environmental DNA and the influence of sunlight. PLoS One 12. https://doi.org/10.1371/journal.pone.0185043

Barnes, M.A., Turner, C.R., Jerde, C.L., Renshaw, M.A., Chadderton, W.L., Lodge, D.M., 2014. Environmental conditions influence eDNA persistence in aquatic systems. Environ Sci Technol 48, 1819–1827. https://doi.org/10.1021/es404734p

Brandão-Dias, P.F.P., Hallack, D.M.C., Snyder, E.D., Tank, J.L., Bolster, D., Volponi, S., Shogren, A.J., Lamberti, G.A., Bibby, K., Egan, S.P., 2023. Particle size influences decay rates of environmental DNA in aquatic systems. Mol Ecol Resour 23, 756–770. https://doi.org/10.1111/1755-0998.13751

Bylemans, J., Furlan, E.M., Gleeson, D.M., Hardy, C.M., Duncan, R.P., 2018. Does Size Matter? An Experimental Evaluation of the Relative Abundance and Decay Rates of Aquatic Environmental DNA. Environ Sci Technol 52, 6408–6416. https://doi.org/10.1021/acs.est.8b01071

Che-Pelicier, A., Hampton, H.G., Sabadel, A.J.M., Thomson Laing, G., Miller, T., Pochon, X., 2025. Release and Degradation of Environmental DNA and RNA From Eels in Aotearoa New Zealand. Environmental DNA 7. https://doi.org/10.1002/edn3.70128

Collins, R.A., Wangensteen, O.S., O’Gorman, E.J., Mariani, S., Sims, D.W., Genner, M.J., 2018. Persistence of environmental DNA in marine systems. Commun Biol 1. https://doi.org/10.1038/s42003-018-0192-6

Cowart, D.A., Murphy, K.R., Cheng, C.H.C., 2018. Metagenomic sequencing of environmental DNA reveals marine faunal assemblages from the West Antarctic Peninsula. Mar Genomics 37, 148–160. https://doi.org/10.1016/j.margen.2017.11.003

Dercksen, J.A., Foppen, J.W., Blom, A., Trimbos, K.B., Gebert, J., Bogaard, T.A., Stancanelli, L.M., 2025. The Impact of Flow Velocity on Environmental DNA Detectability for the Application in River Systems. Environmental DNA 7. https://doi.org/10.1002/edn3.70111

Eichmiller, J.J., Best, S.E., Sorensen, P.W., 2016. Effects of Temperature and Trophic State on Degradation of Environmental DNA in Lake Water. Environ Sci Technol 50, 1859–1867. https://doi.org/10.1021/acs.est.5b05672

Ellis, M.R., Clark, Z.S.R., Treml, E.A., Brown, M.S., Matthews, T.G., Pocklington, J.B., Stafford-Bell, R.E., Bott, N.J., Nai, Y.H., Miller, A.D., Sherman, C.D.H., 2022. Detecting marine pests using environmental DNA and biophysical models. Science of the Total Environment 816. https://doi.org/10.1016/j.scitotenv.2021.151666

Ely, T., Barber, P.H., Man, L., Gold, Z., 2021. Short-lived detection of an introduced vertebrate eDNA signal in a nearshore rocky reef environment. PLoS One 16. https://doi.org/10.1371/journal.pone.0245314

Forsström, T., Vasemägi, A., 2016. Can environmental DNA (eDNA) be used for detection and monitoring of introduced crab species in the Baltic Sea? Mar Pollut Bull 109, 350–355. https://doi.org/10.1016/j.marpolbul.2016.05.054

Holman, L.E., Chng, Y., Rius, M., 2022. How does eDNA decay affect metabarcoding experiments? Environmental DNA 4, 108–116. https://doi.org/10.1002/edn3.201

Jo, T., Arimoto, M., Murakami, H., Masuda, R., Minamoto, T., 2020. Estimating shedding and decay rates of environmental nuclear DNA with relation to water temperature and biomass. Environmental DNA 2, 140–151. https://doi.org/10.1002/edn3.51

Jo, T., Minamoto, T., 2021. Complex interactions between environmental DNA (eDNA) state and water chemistries on eDNA persistence suggested by meta-analyses. Mol Ecol Resour 21, 1490–1503. https://doi.org/10.1111/1755-0998.13354

Jo, T., Murakami, H., Masuda, R., Sakata, M.K., Yamamoto, S., Minamoto, T., 2017. Rapid degradation of longer DNA fragments enables the improved estimation of distribution and biomass using environmental DNA. Mol Ecol Resour 17, e25–e33. https://doi.org/10.1111/1755-0998.12685

Jo, T.S., 2023. Factors affecting biphasic degradation of eDNA released by Japanese jack mackerel (Trachurus japonicus). J Exp Mar Biol Ecol 568. https://doi.org/10.1016/j.jembe.2023.151941

Kirtane, A., Wieczorek, D., Noji, T., Baskin, L., Ober, C., Plosica, R., Chenoweth, A., Lynch, K., Sassoubre, L., 2021. Quantification of Environmental DNA (eDNA) shedding and decay rates for three commercially harvested fish species and comparison between eDNA detection and trawl catches. Environmental DNA 3, 1142–1155. https://doi.org/10.1002/edn3.236

Kutti, T., Johnsen, I.A., Skaar, K.S., Ray, J.L., Husa, V., Dahlgren, T.G., 2020. Quantification of eDNA to Map the Distribution of Cold-Water Coral Reefs. Front Mar Sci 7. https://doi.org/10.3389/fmars.2020.00446

Kwong, S.L.T., Villacorta-Rath, C., Doyle, J., Uthicke, S., 2021. Quantifying Shedding and Degradation Rates of Environmental DNA (eDNA) from Pacific Crown-of-thorns Seastar (Acanthaster cf. Solaris). https://doi.org/10.21203/rs.3.rs-250279/v1

McCartin, L.J., Vohsen, S.A., Ambrose, S.W., Layden, M., McFadden, C.S., Cordes, E.E., McDermott, J.M., Herrera, S., 2022. Temperature Controls eDNA Persistence across Physicochemical Conditions in Seawater. Environ Sci Technol 56, 8629–8639. https://doi.org/10.1021/acs.est.2c01672

Minamoto, T., Fukuda, M., Katsuhara, K.R., Fujiwara, A., Hidaka, S., Yamamoto, S., Takahashi, K., Masuda, R., 2017. Environmental DNA reflects spatial and temporal jellyfish distribution. PLoS One 12. https://doi.org/10.1371/journal.pone.0173073

Murakami, H., Yoon, S., Kasai, A., Minamoto, T., Yamamoto, S., Sakata, M.K., Horiuchi, T., Sawada, H., Kondoh, M., Yamashita, Y., Masuda, R., 2019. Dispersion and degradation of environmental DNA from caged fish in a marine environment. Fisheries Science 85, 327–337. https://doi.org/10.1007/s12562-018-1282-6

Nevers, M.B., Byappanahalli, M.N., Morris, C.C., Shively, D., Przybyla-Kelly, K., Spoljaric, A.M., Dickey, J., Roseman, E.F., 2018. Environmental DNA (eDNA): A tool for quantifying the abundant but elusive round goby (Neogobius melanostomus). PLoS One 13. https://doi.org/10.1371/journal.pone.0191720

Nevers, M.B., Przybyla-Kelly, K., Shively, D., Morris, C.C., Dickey, J., Byappanahalli, M.N., 2020. Influence of sediment and stream transport on detecting a source of environmental DNA. PLoS One 15. https://doi.org/10.1371/journal.pone.0244086

Qian, T., Shan, X., Wang, W., Jin, X., 2022. Effects of Temperature on the Timeliness of eDNA/eRNA: A Case Study of Fenneropenaeus chinensis. Water (Switzerland) 14. https://doi.org/10.3390/w14071155

Saito, T., Doi, H., 2021. Degradation modeling of water environmental DNA: Experiments on multiple DNA sources in pond and seawater. Environmental DNA 3, 850–860. https://doi.org/10.1002/edn3.192

Sassoubre, L.M., Yamahara, K.M., Gardner, L.D., Block, B.A., Boehm, A.B., 2016. Quantification of Environmental DNA (eDNA) Shedding and Decay Rates for Three Marine Fish. Environ Sci Technol 50, 10456–10464. https://doi.org/10.1021/acs.est.6b03114

Scriver, M., Zaiko, A., Pochon, X., von Ammon, U., 2023. Harnessing decay rates for coastal marine biosecurity applications: A review of environmental DNA and RNA fate. Environmental DNA. https://doi.org/10.1002/edn3.405

Seymour, M., Durance, I., Cosby, B.J., Ransom-Jones, E., Deiner, K., Ormerod, S.J., Colbourne, J.K., Wilgar, G., Carvalho, G.R., de Bruyn, M., Edwards, F., Emmett, B.A., Bik, H.M., Creer, S., 2018. Acidity promotes degradation of multi-species environmental DNA in lotic mesocosms. Commun Biol 1. https://doi.org/10.1038/s42003-017-0005-3

Sheehan, R., Knight, I.T., Cangelosi, A., Melendez, A., Phillips, H., Welsbacher, A., Gruwell, M.E., 2024. Quantitative assessment of planktonic AIS eDNA signal duration in Great Lakes harbor water microcosms. Management of Biological Invasions 15, 567–580. https://doi.org/10.3391/mbi.2024.15.4.07

Shogren, A.J., Tank, J.L., Egan, S.P., August, O., Rosi, E.J., Hanrahan, B.R., Renshaw, M.A., Gantz, C.A., Bolster, D., 2018. Water Flow and Biofilm Cover Influence Environmental DNA Detection in Recirculating Streams. Environ Sci Technol 52, 8530–8537. https://doi.org/10.1021/acs.est.8b01822

Sigsgaard, E.E., Nielsen, I.B., Bach, S.S., Lorenzen, E.D., Robinson, D.P., Knudsen, S.W., Pedersen, M.W., Jaidah, M. Al, Orlando, L., Willerslev, E., Møller, P.R., Thomsen, P.F., 2016. Population characteristics of a large whale shark aggregation inferred from seawater environmental DNA. Nat Ecol Evol 1. https://doi.org/10.1038/s41559-016-0004

Snyder, E.D., Tank, J.L., Brandão-Dias, P.F.P., Bibby, K., Shogren, A.J., Bivins, A.W., Peters, B., Curtis, E.M., Bolster, D., Egan, S.P., Lamberti, G.A., 2023. Environmental DNA (eDNA) removal rates in streams differ by particle size under varying substrate and light conditions. Science of the Total Environment 903. https://doi.org/10.1016/j.scitotenv.2023.166469

Thomsen, P.F., Kielgast, J., Iversen, L.L., Møller, P.R., Rasmussen, M., Willerslev, E., 2012. Detection of a Diverse Marine Fish Fauna Using Environmental DNA from Seawater Samples. PLoS One 7, e41732. https://doi.org/10.1371/journal.pone.0041732

Trafford, J.D., Garner, T.W.J., Murrell, D.J., Day, J.J., 2024. Experimental Evidence of Rapidly Decaying Environmental DNA Highlights Infection Risk from Two Major Amphibian Pathogens. Environmental DNA 6. https://doi.org/10.1002/edn3.70051

Weltz, K., Lyle, J.M., Ovenden, J., Morgan, J.A.T., Moreno, D.A., Semmens, J.M., 2017. Application of environmental DNA to detect an endangered marine skate species in the wild. PLoS One 12. https://doi.org/10.1371/journal.pone.0178124

Wood, S.A., Biessy, L., Latchford, J.L., Zaiko, A., von Ammon, U., Audrezet, F., Cristescu, M.E., Pochon, X., 2020. Release and degradation of environmental DNA and RNA in a marine system. Science of the Total Environment 704. https://doi.org/10.1016/j.scitotenv.2019.135314

Yu, X., Zhou, J., Wei, J., Zhang, B., Lu, X., 2022. Temperature May Play a More Important Role in Environmental DNA Decay than Ultraviolet Radiation. Water (Switzerland) 14. https://doi.org/10.3390/w14193178
